# A Novel Superparamagnetic‐Responsive Hydrogel Facilitates Disc Regeneration by Orchestrating Cell Recruitment, Proliferation, and Differentiation within Hostile Inflammatory Niche

**DOI:** 10.1002/advs.202408093

**Published:** 2024-10-07

**Authors:** Borui Xue, Yan Peng, Yongfeng Zhang, Shijie Yang, Yi Zheng, Huiling Hu, Xueli Gao, Beibei Yu, Xue Gao, Shengyou Li, Haining Wu, Teng Ma, Yiming Hao, Yitao Wei, Lingli Guo, Yujie Yang, Zhenguo Wang, Tingfeng Xue, Jin Zhang, Beier Luo, Bing Xia, Jinghui Huang

**Affiliations:** ^1^ Department of Orthopaedics Xijing Hospital The Fourth Military Medical University Xi'an 710032 P. R. China; ^2^ Air Force 986(th) Hospital The Fourth Military Medical University Xi'an 710032 P. R. China; ^3^ College of Advanced Manufacturing Fuzhou University Jinjiang 362200 P. R. China; ^4^ Department of Neurosurgery The Second Affiliated Hospital of Xi'an Jiao Tong University Xi'an 710032 P. R. China; ^5^ School of Ecology and Environment Northwestern Polytechnical University Xi'an 710072 P. R. China; ^6^ College of Chemical Engineering Fuzhou University Xueyuan Road Fuzhou 350108 P. R. China; ^7^ Department of Spinal Surgery Shanghai Changhai Hospital Affiliated to Naval Medical University Shanghai 200433 P. R. China

**Keywords:** intervertebral disc regeneration, mechanical stimulation, nucleus pulposus stem cells, superparamagnetic hydrogel

## Abstract

In situ disc regeneration is a meticulously orchestrated process, which involves cell recruitment, proliferation and differentiation within a local inflammatory niche. Thus far, it remains a challenge to establish a multi‐staged regulatory framework for coordinating these cellular events, therefore leading to unsatisfactory outcome. This study constructs a super paramagnetically‐responsive cellular gel, incorporating superparamagnetic iron oxide nanoparticles (SPIONs) and aptamer‐modified palladium‐hydrogen nanozymes (PdH‐Apt) into a double‐network polyacrylamide/hyaluronic acid (PAAm/HA) hydrogel. The Aptamer DB67 within magnetic hydrogel (Mag‐gel) showed a high affinity for disialoganglioside (GD2), a specific membrane ligand of nucleus pulposus stem cells (NPSCs), to precisely recruit them to the injury site. The Mag‐gel exhibits remarkable sensitivity to a magnetic field (MF), which exerts tunable micro/nano‐scale forces on recruited NPSCs and triggers cytoskeletal remodeling, consequently boosting cell expansion in the early stage. By altering the parameters of MF, the mechanical cues within the hydrogel facilitates differentiation of NPSCs into nucleus pulposus cells to restore disc structure in the later stage. Furthermore, the PdH nanozymes within the Mag‐gel mitigate the harsh inflammatory microenvironment, favoring cell survival and disc regeneration. This study presents a remote and multi‐staged strategy for chronologically regulating endogenous stem cell fate, supporting disc regeneration without invasive procedures.

## Graphical abstract

The superparamagnetic‐responsive hydrogel integrates SPIONs and aptamer‐modified PdH nanozymes to selectively recruit endogenous stem cells and enhance their proliferation and differentiation through adjustable magnetic fields, while also improving the inflammatory microenvironment. This study introduces a remote and multi‐staged strategy for chronologically regulating endogenous stem cell fate in endogenous regeneration.

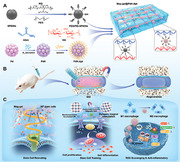

## 1 Introduction

Low back pain (LBP) is highly prevalent, impacting over 80% of the global population and standing as a leading contributor to disability.^[^ ^1^ ^]^ Approximately 40% of reported LBP cases stem from symptomatic intervertebral disc (IVD) degeneration (IDD).^[^ ^2^ ^]^ Palliative treatment for mild IDD includes analgesics, anti‐inflammatory drugs, and physical therapy.^[^ ^3^ ^]^ Conservative therapies have limited effectiveness in delaying or preventing the IDD, providing only temporary relief from symptoms and limited functional improvement. Surgical procedures such as discectomy and spinal fusion are considered the last resort for IDD. However, these highly‐invasive surgeries pose the risk of altering the biomechanics of the spine and hastening the degeneration of nearby discs, ultimately resulting in a pyrrhic victory.^[^ ^4^ ^]^ The IVD comprises a central nucleus pulposus (NP), encircled by the annulus fibrosus (AF) and delimited from adjacent vertebral bodies by thin cartilaginous end plates.^[^ ^5^ ^]^ Numerous studies have shown that NP cells (NPCs) dysfunction is the primary cause of IDD;^[^ ^6^, ^7^ ^]^ therefore, it is desirable to develop a new therapeutic approach that can effectively restore the biological functions of NPCs.

The homeostasis of the IVD depends heavily on the proper functioning of NPCs, particularly in regards to their maintenance and secretion of the extracellular matrix (ECM). NPCs undergo biological changes that lead to IDD, including the conversion of cell types, an observed reduction in functional cell count,^[^ ^4^ ^]^ and an elevation in cellular aging.^[^ ^8^ ^]^ Therefore, to reverse IDD, it is essential to reintroduce NPCs and trigger their physiological roles in the IVD. Exogenous stem cell transplantation has emerged as a promising therapeutic strategy, given its wide availability and the potential for multilineage differentiation.^[^ ^9^, ^10^ ^]^ However, the use of exogenous stem cells is hindered by potential immune rejection, pathogen transmission, tumorigenesis, and loss of cell viability or phenotype.^[^ ^11^ ^]^ Furthermore, local conditions within the IVD, such as overloading, nutrient deficiency, and low oxygen levels, greatly restrict the survival of the transplanted stem cells, leading to compromised outcomes.^[^ ^12^, ^13^ ^]^ Therefore, we investigated strategies to surmount these difficulties in this study.

The capacity for regeneration without scarring is limited to the early developmental stages in mammals and diminishes gradually with growth. Endogenous tissue regeneration post‐injury is mediated by locally residing tissue‐specific stem cells.^[^ ^14^ ^]^ Recently, native stem cell niches have been discovered near the epiphyseal plate in the perichondria region and in the outer zone of the annulus fibrosus in IVD.^[^ ^15^ ^]^ These niches act as reservoirs for endogenous nucleus pulposus stem cells (NPSCs), which have a strong pro‐repair capacity; these stem cells represent a potential strategy for reversing endogenous degeneration.^[^ ^16^, ^17^ ^]^ However, mobilizing endogenous NPSCs to orchestrate disc regeneration remains challenging. There are three main obstacles to overcome. First, the modalities to attract reparative stem cells from the peripheral niches to the central region have not yet been defined. Second, the quantity and biological activity of the NPSCs within the IVD markedly decrease with age and degeneration,^[^ ^18^, ^19^ ^]^ highlighting the need to promote expansion of these limited stem cell populations to compensate for age‐related depletion. Following cell expansion, appropriate signals are required to trigger NPSCs phenotypic differentiation into terminal NPCs. Finally, the IVD is an avascular organ that is immune‐privileged. It facilitates a strong inflammatory response through various downstream cascade reactions during degeneration. The harsh inflammatory environment of IDD drastically compromises stem cell survival and limits their repair capacity.^[^ ^20^, ^21^ ^]^ As such, a comprehensive temporal strategy that can recruit endogenous NPSCs to the central region, support their survival and differentiation, and simultaneously ameliorate harsh local inflammatory environments has great potential for orchestrating biological disc regeneration.

**Scheme 1:**
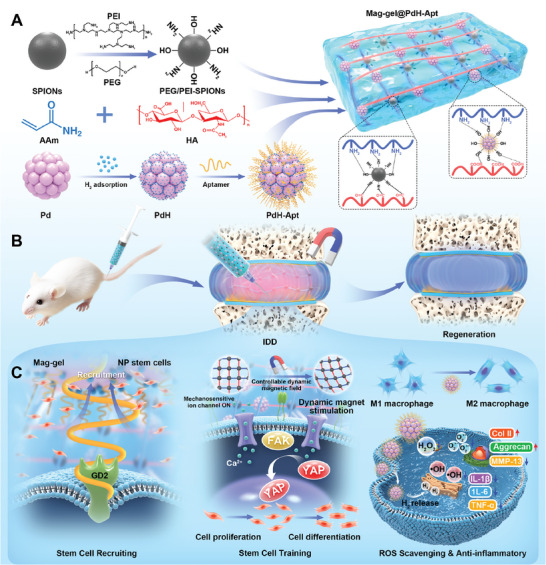
Schematic illustration of fabrication of superparamagnetic hydrogel and its role in enhancing in situ IVD regeneration. A) Fabrication of superparamagnetic hydrogel. B) Hydrogel is transplanted into degenerated IVD, and then stimulates with MF in vitro to promote NP regeneration. C) Magnetotactic hydrogel promotes NP repair by recruiting endogenous stem cells, initiating stem cell training, and suppressing ROS and inflammation.

In this study, a superparamagnetic‐responsive hydrogel (Mag‐gel@PdH‐Apt) was devised by incorporating polyethylene glycol/polyethyleneimine‐modified superparamagnetic nanoparticles (SPIONs) and aptamer‐modified palladium‐hydrogen nanozymes (PdH‐Apt) into a double‐network polyacrylamide/hyaluronic acid (PAAm/HA) hydrogel (**Scheme** **1**A). This hydrogel integrated both chemical and physical signals to recruit endogenous NPSCs and remotely control their cellular behavior, thereby orchestrating IVD regeneration. The Mag‐gel contained aptamer DB67, which had a high affinity for disialoganglioside (GD2), a specific membrane ligand of NPSCs.^[^ ^16^, ^22^ ^]^ This allowed for precise attraction and recruitment of endogenous NPSCs from the peripheral to the central region. Furthermore, the hydrogel exhibited remarkable sensitivity to wireless magnetic field (MF), which provided tunable mechanical cues and served as a mechanical‐training platform to apply forces on recruited NPSCs for promoting their proliferation and expansion. Utilizing the convenience of MF as an external stimulus controller, we noninvasively provided mechanical cues to NPSCs within the Mag‐gel, triggered their differentiation into NPCs, and realized a restoration of the NP architecture. Finally, the remarkable anti‐inflammatory and anti‐oxidative capabilities of the palladium hydrogen (PdH) offered an opportunity to overcome the hostile inflammatory environment during IDD, and paved the way for disc regeneration by orchestrating endogenous stem cells (Scheme 1B,C). This mechanical‐training platform integrated physical and chemical cues to provide a remotely‐controllable synthetic strategy for disc regeneration with temporal precision.

## 2 Results

### 2.1 Synthesis and Characterization of Superparamagnetic Hydrogels

**Figure 1:**
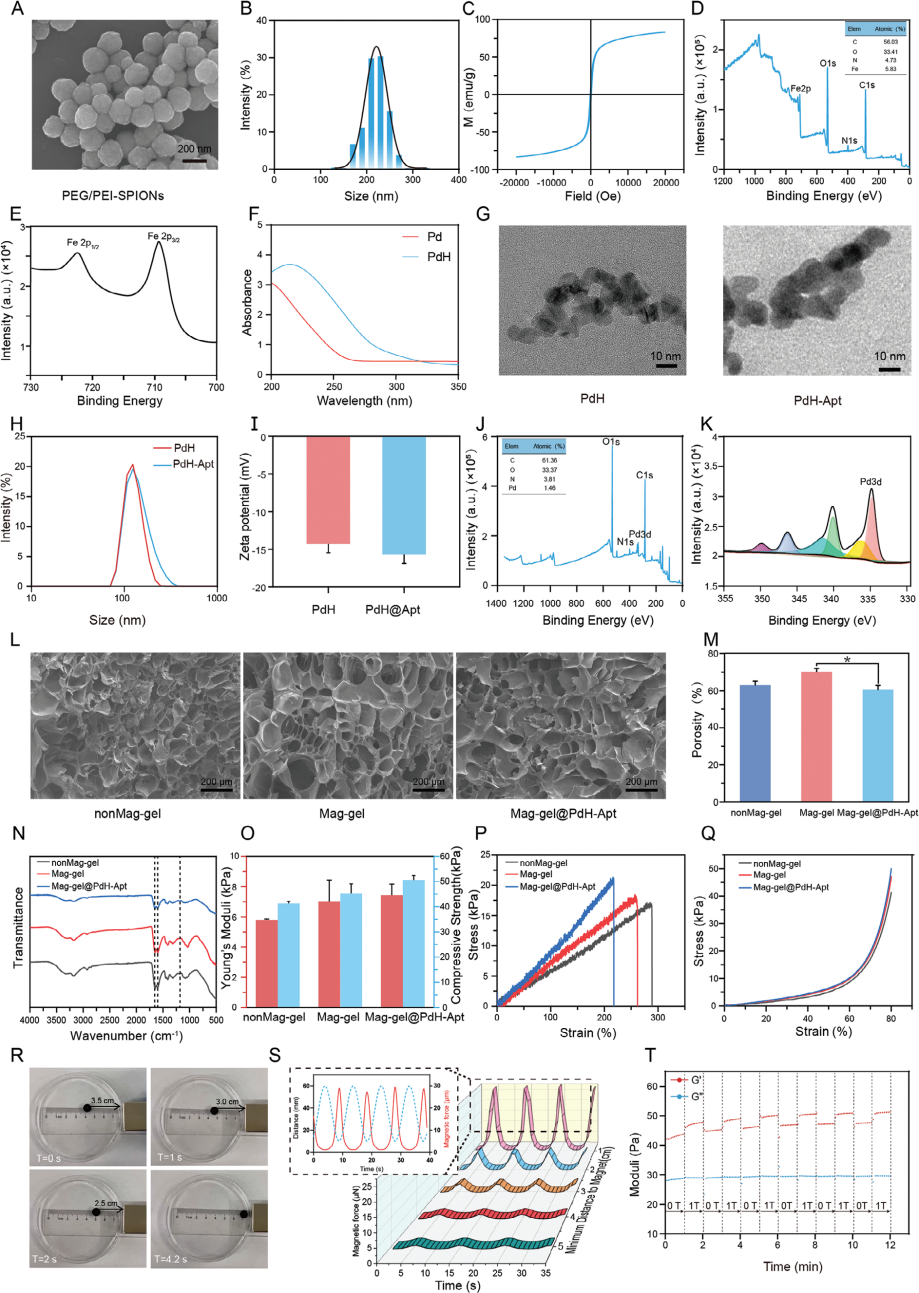
Characterization of Mag‐gel@PdH‐Apt. A) Transmission electron microscopy (TEM) visualization of synthesized PEG/PEI‐SPIONs. B) Particle size distribution of PEG/PEI‐SPIONs calculated from TEM images. C) Hysteresis behavior observed in PEG/PEI‐SPIONs at standard room conditions. D) High‐resolution XPS of PEG/PEI‐SPIONs. E) The high‐resolution XPS spectra of Fe 2p were obtained for PEG/PEI‐SPIONs. F) UV–vis–NIR spectra of Pd and PdH. G) TEM images of fabricated PdH and PdH‐Apt. H) Particle size distributions of PdH and PdH‐Apt. I) Zeta potentials of PdH and PdH‐Apt. J) High‐resolution XPS spectrum of PdH‐Apt. K) High‐resolution XPS spectrum of Pd 3d for PdH‐Apt. L) Microstructures of nonMag‐gel, Mag‐gel, and Mag‐gel@PdH‐Apt observed using SEM. M) Porosities of nonMag‐gel, Mag‐gel, and Mag‐gel@PdH‐Apt. N) FTIR spectra of nonMag‐gel, Mag‐gel, and Mag‐gel@PdH‐Apt. O) Young's moduli of nonMag‐gel, Mag‐gel, and Mag‐gel@PdH‐Apt. P) Compression stress‐strain curves of nonMag‐gel, Mag‐gel, and Mag‐gel@PdH‐Apt. Q) Tensile stress–strain curves of nonMag‐gel, Mag‐gel, and Mag‐gel@PdH‐Apt. R) Time‐lapse imaging of magnetic hydrogels attracted by permanent magnets. S) Profile of magnetic force exerted on the Mag‐gel@PdH‐Apt by a dynamic magnetic force apparatus, with the magnet moving within a range of 10 to 50 mm from the hydrogel. T) Magnetorheological characterization of Mag‐gel@PdH‐Apt. Date are presented as mean ± standard deviation (SD) (*n* = 3, *P < 0.05).

Synthesizing superparamagnetic hydrogels involved combining polyethyleneglycol/polyethyleneimine modified superparamagnetic nanoparticles (PEG/PEI‐SPIONs), PAAm, HA, and PdH‐Apt as primary components. Cross‐linkers such ase, *N, N, N’, N’*‐tetramethylenediamine (TEMED) and *N, N’*‐methylenebis‐(acrylamide) (MBAA), along with the initiator ammonium persulfate (APS), were utilized. These constituents are commonly employed in regenerative medicine applications.^[^ ^23^ ^]^ Upon addition of the initiator (APS) to the pre‐polymer solution, the PEG/PEI‐SPIONs@PdH‐Apt@PAAm/HA hydrogel underwent complete cross‐linking within 10 min. This cross‐linking process involved both covalent and noncovalent bond formations. The synthesized PEG/PEI‐SPIONs, with a mean size of 219.7 ± 26.2 nm (**Figure** **1**A,B). The magnetization of the PEG/PEI‐SPIONs was 83.16 emu g^−1^ (Figure 1C). Full‐scan spectra were obtained for the PEG/PEI‐SPIONs, and the X‐ray photoelectron spectra (XPS) showed four characteristic peaks: C 1s, N 1s, O 1s, and Fe 2p, as shown in Figure 1D. The dominant peak maxima for Fe 2p_1/2_ and Fe 2p_3/2_ were identified at 722.4 and 709.2 eV, respectively, as displayed in Figure 1E. SPIONs were successfully encapsulated with PEG/PEI to improve their stability, with the structural and functional integrity of the PEG/PEI‐SPIONs were well maintained.

PdH nanozymes have exhibited multienzyme activity and have been commonly used in therapies for atherosclerosis and Alzheimer's disease.^[^ ^24^, ^25^ ^]^ Aptamer‐functionalized nanozymes have been developed as targeted therapies for tumors and regeneration medicine.^[^ ^26^, ^27^ ^]^ In this study, we developed aptamer nanoplatforms bridged with PdH nanozymes for targeted recruitment and autocatalytic therapies. The successful formation of PdH and the desirable H_2_ absorption features of the Pd nanocrystals were demonstrated by a significant increase in the ultraviolet‐visible (UV–vis) intensity of PdH after being loaded with H_2_ (Figure 1F). The PdH and PdH‐Apt exhibited a cubic morphology with uniform dimensions, facilitating efficient H_2_ containment, transport, and discharge (Figure 1G). The diameter of the PdH was ≈196.6 ± 29.4 nm according to dynamic light scattering, whereas that of the PdH‐Apt was ≈214.9 ± 48.1 nm (Figure 1H). Zeta potentials of the PdH and PdH‐Apt were −14.2 and −15.8 mV, respectively (Figure 1I). We also analyzed the full‐scan XPS of the PdH‐Apt and identified four distinct peaks: C1s, N1s, O1s, and Pd3d (Figure 1J). Figure 1K shows that the binding energy value for the peak maximum of Pd3d was estimated at 346.78 eV, indicating a successful bridging of the aptamers to the nanozymes.

The study investigated the use of three hydrogels: nonMag‐gel, Mag‐gel, and Mag‐gel@PdH‐Apt, which were composed of PAAm/HA, PEG/PEI‐SPIONs@PAAm/HA, and PEG/PEI‐SPIONs@PdH‐Apt@PAAm/HA, respectively. All of the hydrogels had highly porous 3D structures that enabled NPSC attachment and extension (Figure 1L). The porosities and average pore diameters of the nonMag‐gel, Mag‐gel, and Mag‐gel@PdH‐Apt samples were ≈62.95 ± 2.13% and 43.26 ± 17.28 µm, 70.20% ± 1.74% and 48.00 ± 22.88 µm, and 60.56% ± 2.23% and 42.59 ± 18.07 µm, respectively. These results indicated that the incorporation of PEG/PEI‐SPIONs and PdH‐Apt had a negligible impact on the pore architecture (Figure 1M; Figure S1, Supporting Information). Energy‐dispersive spectrometry (EDS) and the corresponding elemental mapping data provided supplementary validation for the successful binding of the SPIONs to the PdH‐Apt (Figure S2, Supporting Information). Analysis using Fourier transform infrared (FTIR) spectroscopy revealed specific absorption bands at 1651, 1530, and 1319 cm^−1^, corresponding to amide I, amide II, and amide III, respectively. This indicated the establishment of a dual‐network PAAm/HA cross‐linked hydrogel. The signature peaks were unchanged following the introduction of SPIONs or PdH‐Apt (Figure 1N).

Because the mechanical properties of scaffolds are essential elements for cell activity and disc regeneration,^[^ ^28^ ^]^ the mechanical behavior of the hydrogels was assessed utilizing a universal material testing machine. The elastic modulus of the Mag‐gel@PdH‐Apt was 7.44 ± 0.73 kPa, which was similar to that of healthy IVD tissues (Figure 1O).^[^ ^29^ ^]^ The Mag‐gel@PdH‐Apt showed higher tensile and compressive strengths of 22.00 ± 0.75 and 50.6 ± 1.76 kPa, respectively, compared to the nonMag‐gel and Mag‐gel samples (Figure 1P–Q; Figure S3, Supporting Information). This mechanical improvement primarily stemmed from the creation of a hybrid‐cross‐linked dual architecture, facilitated by the incorporation of SPIONs and PdH‐Apt. Mechanical stability and the reciprocating tensile and compressive properties were assessed by subjecting the material to successive loading–unloading cycles for 10 iterations, each at an 80% compressive strain. (Figure S4, Supporting Information).

Concentrations of the PEG/PEI‐SPIONs directly influenced the magnetism of the Mag‐gel@PdH‐Apt, as shown in Figure S5 (Supporting Information). The magnetism exhibited by the hydrogel was closely related to the concentrations of nanoparticles. As shown in Figure 1R, Figure S6 (Supporting Information), and Video S1 (Supporting Information), the superparamagnetic characteristic of the Mag‐gel@PdH‐Apt was detected upon exposure to a magnetic field. To provide dynamic mechanical stimulation, a permanent magnet (400 mT) was fixed to an electric actuator to apply MF to NPSCs (2 h per day for 7 days). This dynamic magnetic attraction/release on Mag‐gel@PdH‐Apt established a dynamic microenvironment with rhythmic mechanical cues to train NPSCs. The force intensity was modulated by altering the separation between the hydrogel and magnet as illustrated in Figure 1S. According to previous studies, different stimulation frequencies lead to distinct cell fates. Lower frequencies effectively enhance the proliferation of mesenchymal stem cells, with negligible effects on cell stemness.^[^ ^30^ ^]^ Conversely, increasing the stimulation frequency to 0.5 Hz could trigger stem cells to differentiate toward chondrogenesis.^[^ ^31^, ^32^ ^]^ Therefore, we utilized a 0.1 Hz frequency for stem cell expansion (Figure S7, Table S1, and Video S2, Supporting Information), while increasing the frequency to 0.5 Hz to induce stem cell differentiation. A rheological test was conducted on the Mag‐gel@PdH‐Apt to investigate the forces produced by the SPIONs as it was exposed to the magnetic field. The addition of MF at 1 T resulted in an approximate increase of 3.63 ± 0.43 Pa in the storage modulus of the Mag‐gel, with no significant change in the loss modulus per minute (Figure 1T). The swelling ratio of Mag‐gel@PdH‐Apt increased by 4.71 times after being immersed in distilled water for 72 h. This indicated a strong affinity for water and an ability to retain water (Figure S8a, Supporting Information). By day 28, the remaining weight percentages for the nonMag‐gel, Mag‐gel, and Mag‐gel@PdH‐Apt were 48.00 ± 1.45%, 43.95 ± 2.05%, and 57.91 ± 2.50%, respectively. This indicates a gradual degradation of the hydrogels, leading to the loss of their organized 3D structure, as depicted in Figure S8b (Supporting Information). As the hydrogels degraded, their structure loosened and mass decreased, creating ample space for NP regeneration.^[^ ^33^ ^]^ Instead of physical blending SPIONs into the hydrogel, which might lead to leakage, they were grafted onto PAAm and HA through the formation of chemical bonds. We quantified the percentage of fluorescent SPIONs in the solution surrounding the hydrogel using flow cytometry. We found negligible leakage of magnetic SPIONs from the magnetic hydrogel (<1%), regardless of the application of MF. This ensures that even during hydrogel degradation, SPIONs remain stably encapsulated within the hydrogel without significant leakage (Figure S9, Supporting Information). One‐month post‐degradation, we exposed the Mag‐gel@PdH‐Apt to MF and observed a decrease in both retained storage and loss moduli. However, the hydrogel still exhibited moduli changes in response to MF, ensuring long‐term superparamagnetic properties and allowing for magnetic mechanical training of cells (Figure S10, Supporting Information). PdH‐Apt primarily formed weaker physical cross‐links with HA or PAAm via electrostatic attraction and hydrogen bonding. Therefore, PdH‐Apt might undergo sustained release upon degradation of the hydrogel. After one month of degradation, we collected the solution surrounding the Mag‐gel@PdH‐Apt hydrogel for flow cytometry analysis to quantify the percentage of fluorescent PdH‐Apt. We found that compared to the negative control group (solution surrounding non‐degraded hydrogel), the composite hydrogel released 46.2% and 46% of PdH‐Apt with or without MF, respectively (Figure S11, Supporting Information). This indicated sustained release of PdH‐Apt upon hydrogel degradation, with nearly half of the PdH retained in the hydrogel, ensuring continued release and biological efficacy.

### 2.2 DB67 Aptamer‐Functionalized Mag‐Gel Recruited NPSCs

**Figure 2:**
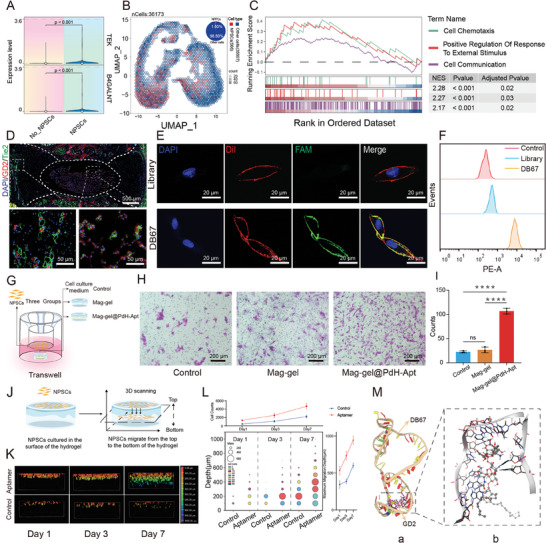
In vitro assessment of aptamer targeting and recruitment ability for NPSCs. A) Violin plots of *TEK* and *B4GALNT* genes in NPSCs and non‐NPSCs. B) Uniform manifold approximation and projection (UMAP) visualization showing gene expression level of *TEK*, *GB4GALNT*, and other cell types from NPSCs inside NP tissues. C) GSEA of NPSC marker genes. D) Immunofluorescence staining of GD2 and Tie2 expression in the IVD. E) Confocal microscopy images demonstrating colocalization of Aptamer DB67‐FAM binding on NPSCs. Cell membranes were stained with Dil. F) Flow cytometric analysis of NPSCs after incubation with Cy3‐labeled DB67. G) Schematic depiction of transwell assay for studying the impact of DB67 on NPSCs migration. H) Representative images of NPSCs migrating from each group. I) Statistical data from transwell assay (*n* = 3, ****P < 0.0001). J) Schematic illustration of NPSCs recruitment based on hydrogel with or without aptamer. K) Confocal observation of top‐to‐bottom cell recruitment at 1, 3, and 7 days after implantation of NPSCs on hydrogel surface. L) Statistics of maximum cell penetration depth of hydrogels and number of cells in different layers. M) Evaluation of DB67 core region for GD2 recognition and binding (a. binding complex of DB67 and GD2; b. 3D diagram of interfacial interaction between GD2 and DB67. C12, G19, A21, C22, A24, and A25 formed polar interactions). All statistical data are presented as mean ± SD.

The presence of NPSC clusters in the nucleus pulposus was confirmed by a single‐cell RNA‐seq database (GSE165722). Two surface markers were identified in NPSCs, including Tie2 (tyrosine kinase with immunoglobulin‐like and EGF‐like domains 2) and GD2, which are encoded by TEK (Tie2 receptor tyrosine kinase) gene and B4GALNT (β−1,4‐N‐Acetyl‐Galactosaminyltransferase 1) gene, respectively (**Figure** **2**A). Within the IVD, NPSCs *co*‐expressing TEK and B4GALNT1 accounted for 1.5% of the total cell population (Figure 2B). Gene set enrichment analysis (GSEA) showed an enrichment in functions associated with cell chemotaxis, positive regulation of response to external stimuli, and cell communication in NPSCs (Figure 2C). We then validated the expressions of Tie2 and GD2 in IVD tissues using immunohistochemical staining. Both Tie2 and GD2 were found in NPSCs in the NP region as well as in native stem cell niches (Figure 2D). We utilized a previously described monoclonal technique to isolate NPSCs from the IVD of rat caudal.^[^ ^34^ ^]^ Subsequently, cell colonies were collected, and the profile of cell surface markers was examined using flow cytometric analysis (Figure S12, Supporting Information). We found that the NPSCs did not express the typical NP marker CD24, but showed high expressions of both GD2 and Tie2. This suggested the successful isolation of highly‐purified NPSCs from NP‐derived cells. Before using the hydrogels, the biological activity of the NPSCs encapsulated within the 3D hydrogels was assessed. Fluorescence imaging of Live/Dead assays after 72 h *co*‐culture revealed minimal presence of dead cells when SPIONs and PdH‐Apt were incorporated into the hydrogels, indicating the high biocompatibility of these hydrogels components (Figure S13, Supporting Information).

Aptamers, comprising single‐stranded DNA or RNA, possess the capability to selectively recognize and bind to molecular targets.^[^ ^35^, ^36^ ^]^ Studies have shown that aptamers can recruit stem cells for bone and cartilage repairs.^[^ ^37^, ^38^, ^39^ ^]^ DB67, a short oligonucleotide, was developed to bind selectively to its specific target GD2 (Table S2, Supporting Information).^[^ ^22^ ^]^ The study began by examining the specificity and affinity of DB67 for anchoring NPSCs. After a 30‐min *co*‐incubation period, the DB67‐FAM@NPSCs were observed with confocal microscopy. CM‐DiI, which emitted red fluorescence, was used to label the cell membranes of the NPSCs. DB67‐FAM displayed a green fluorescence in the cell membrane. The overlaid image showed that the red and green fluorescent signals were colocalized, indicating that DB67 was anchored to the cell membrane of the NPSCs (Figure 2E). Flow cytometry confirmed the targeting specificity of DB67 toward the NPSCs (Figure 2F). DB67 demonstrated a stronger binding affinity to GD2 than did either the random DNA library or the control group. These data verified that DB67 had a stronger affinity for the NPSCs, enabling selective recognition and binding to NPSCs within the IVD.

The recruitment and migration of NPSCs to injury sites are crucial for IVD regeneration. To investigate the effects of DB67 on the recruitment and migratory abilities of NPSCs, we conducted a transwell assay (Figure 2G). After a 24 h culture period, the number of NPSCs that migrated toward the DB67 aptamer exhibited a marked increase compared to both the control and Mag‐gel groups (Figure 2H–I). The control and Mag‐gel groups showed no significant increase in the quantity of migratory cells. To delve deeper into the impact of aptamer DB67 on NPSCs recruitment and migration within the 3D hydrogel, the NPSCs were initially seeded onto its upper surface (Figure 2J). After 1, 3, and 7 days of culture, we measured the maximum depth and quantity of migrated cells. Over time, the number and depth of migrating cells were increased in all the groups, especially for the hydrogel containing DB67, which exhibited a significant increase in the maximum migration depth and number of cells per layer compared with the control group (Figure 2K,L). To explore the key molecular region of DB67 that recognized GD2, we used molecular docking to simulate the interactions between GD2 and DB67. Figure 2M‐a shows the 3D configuration of DB67. DB67 primarily comprised several stem ring structures. Upon docking with GD2, the results of the docking position suggested that the binding was specifically associated with the structure of the nucleic acids and exhibited a certain level of consistency. The active binding sites of DB67, including C12, G19, A21, C22, A24, and A25, established polar contacts for targeting recognition (Figure 2M‐b). These findings indicated that DB67 had a particular binding affinity for the NPSCs and effectively promoted their migration toward the targeting location.

### 2.3 Magneto‐Mechanical Stimulation Enhanced Proliferation of NPSCs

**Figure 3:**
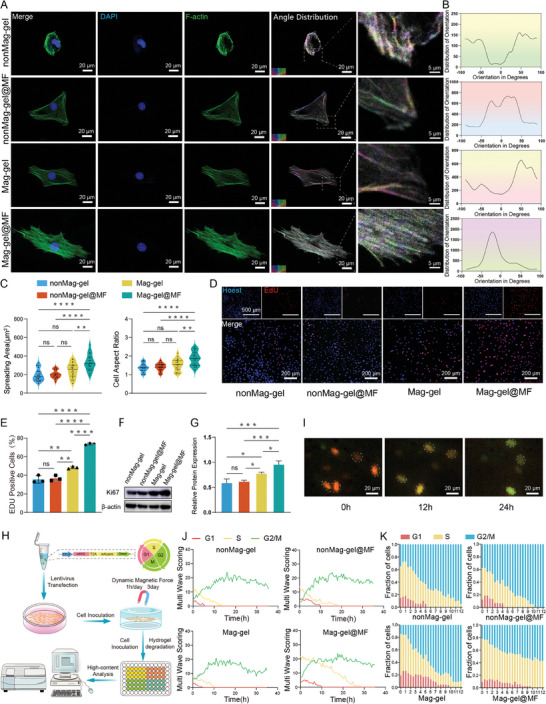
In vitro assessment of the impact of mechanical training on cellular cytoskeletal remodeling and proliferation. A) Confocal immunofluorescence and color‐mapped images of cell morphology and F‐actin polymerization in NPSCs under different culture conditions. B) Angle distribution of F‐actin. C) Violin plots depicting the spreading areas and aspect ratios of NPSCs across different conditions (*n* = 20, **P < 0.01; and ****P < 0.0001). D) Representative images of EdU staining. E) Quantification of EdU‐positive cell rates among various treatment groups (*n* = 3, **P < 0.01; ****P < 0.0001). F,G) Western blot (WB) analysis and quantitation of Ki67 protein levels among different groups (*n* = 3, *P < 0.05; ***P < 0.001). H) Scheme for real‐time single‐cell cyclic dynamics detection using fluorescent ubiquitination‐based cell cycle indicator (FUCCI) system. I) Fluorescence delay imaging of cell cycle alterations in Fucci‐expressing NPSCs after different treatments. J) Fluorescence multiwave scorings of red, yellow, and green cells after different treatments from three fields of view per timepoint. K) Fractions of NPSCs in different cell cycles after different treatments. All statistical data are presented as mean ± SD.

Cell morphology and the development of stress fiber are intricately intertwined with the destiny of cells.^[^ ^40^ ^]^ In response to mechanical cues, these cells undergo reorganization of the F‐actin network and generate cytoskeletal tension, initiating a signaling cascade in turn.^[^ ^41^ ^]^ Hence, our research delved into how magneto‐mechanical actuation influences cytoskeleton formation in NPSCs. According to previous studies, low frequencies MF effectively enhance the proliferation of mesenchymal stem cells, with negligible effects on cell stemness.^[^ ^30^ ^]^ Therefore, we utilized a 0.1 Hz frequency for stem cell expansion in the early stage. NPSCs were cultured in hydrogels under four experimental conditions to observe their cytoskeletal organization: 1) nonMag‐gel group, nonMag‐gel without MF; 2) nonMag‐gel@MF group, nonMag‐gel exposed to MF; 3) Mag‐gel group, Mag‐gel without MF; and 4) Mag‐gel@MF group, Mag‐gel exposed to MF. The NPSCs exhibited a disorganized arrangement of actin fibers in the nonMag‐gel, nonMag‐gel@MF, and Mag‐gel groups. In contrast, the actin fibers were aligned in a specific direction in the Mag‐gel@MF group (**Figure** **3**A,B). In the Mag‐gel@MF group, actin assembly occurred mainly in the filamentous network, resulting in notably broader actin fronts in contrast to the other groups. Furthermore, cells generally remodeled their cytoskeleton by modulating intracellular stress or tension in reaction to mechanical stimuli from the environment. The distribution of F‐actin correlated with cytoskeletal integrity, suggesting a decrease in cytoskeletal tension within the nonMg‐gel group. However, a narrower distribution of actin alignment was detected in the Mag‐gel@MF treatment group, suggesting elevated cytoskeletal tension attributable to magneto‐mechanical actuation. Moreover, NPSCs in the Mag‐gel@MF group exhibited elongated and polygonal shapes, with a significant increase in cell area and cell aspect ratio compared to those of the other groups (Figure 3C).

The effect of magneto‐mechanical stimulation on cell proliferation was investigated by considering orientation of the actin filament and its influence on the stem cell density. Ethynyl‐*2*‐deoxyuridine (EdU) was used to label proliferative NPSCs in each group. The Mag‐gel@MF group exhibited the greatest proportion of EdU‐positive cells (73.63 ± 0.94%), followed by the Mag‐gel (48.30 ±1.26%), nonMag‐gel@MF (36.68 ± 2.97%), and nonMag‐gel (35.50 ± 4.10%) groups (Figure 3D,E). Consistent with the EdU assay results, the Mag‐gel@MF group exhibited a meaningful elevation in Ki67 expression. (Figure 3F,G).^[^ ^42^ ^]^ We used a fluorescence ubiquitination‐based cell cycle indicator (FUCCI) system to examine single‐cell spatiotemporal dynamics with high resolution after magneto‐mechanical actuation.^[^ ^42^, ^43^ ^]^ The cell populations emitting red, yellow, and green lights corresponded to cells in G1, G1/S, and S/G2‐M phases, respectively (Figure 3H,I). As the overall cell counts increased over time, the proportions of red, yellow, and green cells in each group exhibited reciprocally oscillating profiles. The initial yellow fraction accumulated for 30 h in the Mag‐gel@MF group, demonstrating a marked elevation compared to that observed in the other groups (Figure 3J). Furthermore, the Mag‐gel@MF group showed a higher proportion of yellow cells (Figure 3K), indicating that magneto‐mechanical actuation facilitated the G1‐S phase transition in the cell cycle, thereby promoting cell proliferation.

### 2.4 Magneto‐Mechanical Stimulation Promoted NPSC Proliferation by Modulating Calcium Channels

**Figure 4:**
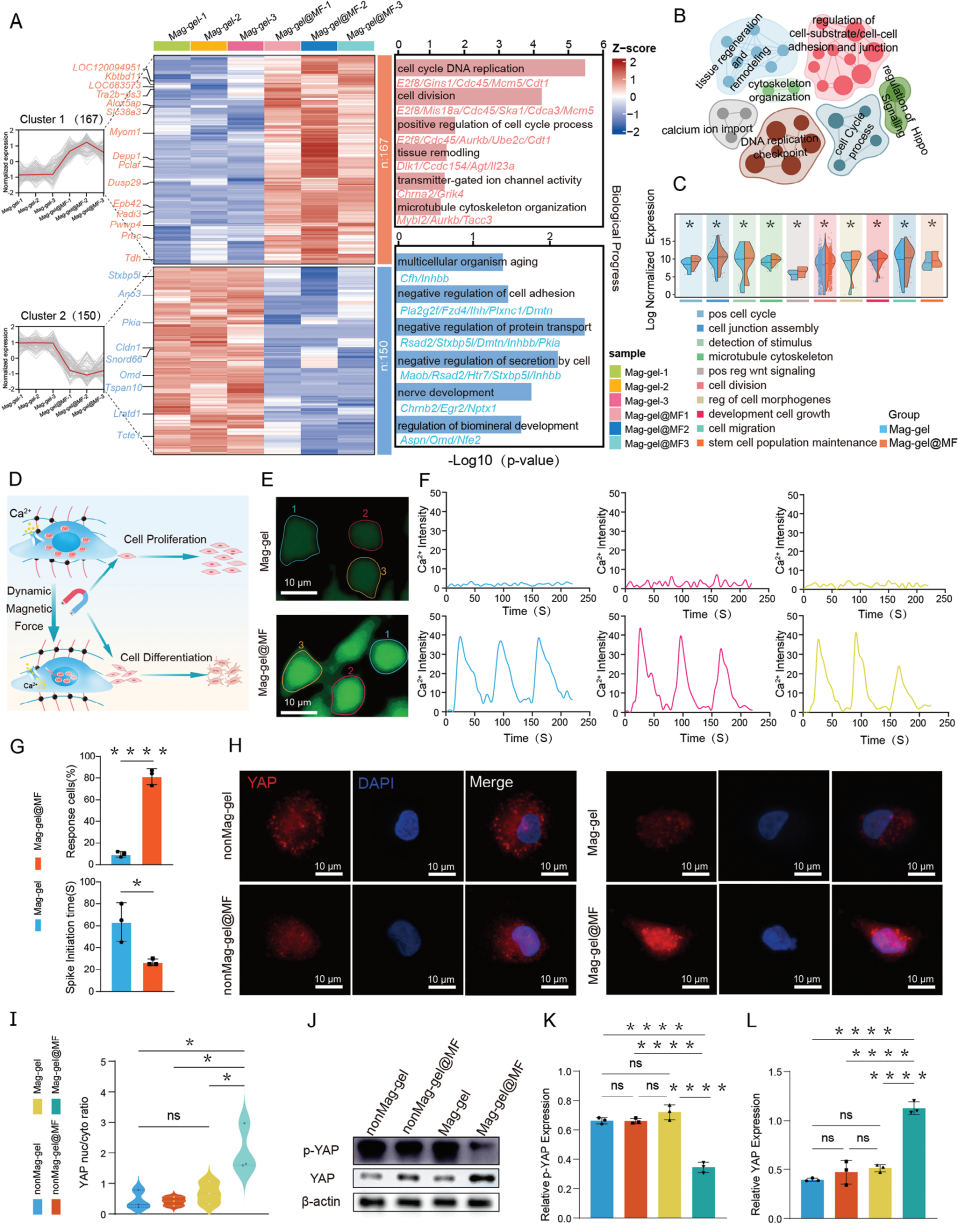
Mechanical stimulation‐regulated mechanism of NPSC proliferation. A) RNA‐seq analyses of significantly regulated pathways in Mag‐gel@MF versus Mag‐gel. Left: Number and trend of up‐regulated and down‐regulated genes in Mag‐gel@MF versus Mag‐gel. Middle: Heatmap of DEG expression levels between different groups. Right: Functional enrichment analysis and annotation for up‐ and down‐regulated genes. B) GSEA for DEGs of Mag‐gel@MF versus Mag‐gel. C) Bean plot showing expression of up‐regulated genes associated with critical pathways in Mag‐gel@MF group. D) Schematic illustration of magneto‐mechanical training directing NPSC proliferation and differentiation by opening calcium channels and promoting YAP nuclear translocation. E,F) Representative images of real‐time Ca^2+^ imaging and quantitative data of Ca^2+^ fluorescence intensity of differently treated NPSCs (*n* = 3). G) Comparison of number of calcium spikes and initiation calcium spikes in NPSCs under different treatments (*n* = 3, *P < 0.05, ****P < 0.0001). H) Immunofluorescence images of YAP. I) Quantitative assessment of YAP localization in the nucleus (*n* = 3, *P < 0.05). J–L) Western blot analysis and quantification of YAP and phosphorylated‐YAP levels across various treatments (*n* = 3, ****P < 0.0001). All statistical data are presented as mean ± SD.

To clarify the inherent mechanism of mechanical stimulation in changing cell fate, we conducted a high‐throughput transcriptomic analysis. Contrasting the Mag‐gel with the Mag‐gel@MF group, 317 genes exhibited significant differential expression (|log2(FC)| > 1, *P <* 0.05), consisting of 167 up‐regulated and 150 down‐regulated differentially expressed genes (DEGs) (**Figure** **4**A). Gene Ontology (GO) analysis revealed that the DEGs were primarily involved in cell cycle DNA replication (e.g., E2f8, Gins1, Cdc45), cell division (e.g., Mis18a, Ska1, Mcm5), transmitter‐gated ion channel activity (e.g., Chrna2, Grik4), and the microtubule cytoskeleton organization (e.g., Mybl2, Aurkb, Tacc3). GSEA, considering all the genes that showed differential expression, uncovered a noteworthy enhancement in crucial biological processes following the administration of the Mag‐gel@MF treatment. These processes encompass tissue regeneration and remodeling, calcium ion import, DNA replication checkpoints, cell cycle progression, cytoskeleton organization, regulation of Hippo signaling, and adjustment of cell‐substrate/cell‐cell adhesion and junctions (Figure 4B). By comparing the overall upregulation of key biological processes, we emphasized the multifaceted impact of Mag‐gel@MF treatment on cellular functionality and regulatory pathways. The Mag‐gel@MF group showed higher expression of enriched pathway genes compared with the Mag‐gel group (Figure 4C), suggesting that magneto‐mechanical stimulation has the potential to enhance cellular proliferation through skeletal remodeling and the activation of calcium channels.

To understand the impact of mechanical forces on the function of mechanosensitive ion channels, we investigated the effects of magneto‐mechanical stimulation on the intracellular Ca^2+^ dynamics in NPSCs in vitro. Our results revealed unique Ca^2+^ oscillations with robust repetitive Ca^2+^ spikes in NPSCs in response to steady magneto‐mechanical forces. In contrast, NPSCs in the Mag‐gel showed a weakened Ca^2+^ oscillatory response, with only a few weak Ca^2+^ spikes that had not been exposed to the magnetic field (Figure 4D–F). Quantitative analyses showed that NPSCs in the Mag‐gel@MF group exhibited a substantial increase in the proportion of Ca^2+^‐responsive cells and a decrease in the Ca^2+^ spike initiation time compared to those of the Mag‐gel group (Figure 4G). These findings indicated that dynamic magneto‐mechanical forces activated mechanosensitive ion channels in NPSCs, leading to increased calcium signaling. In addition, mechanical cues and cytoskeletal tension triggered the cytoplasmic‐nuclear translocation of Yes‐associated protein (YAP) in the Mag‐gel@MF group (Figure 4H,I), which acts as a transcription factor facilitating cellular responses to mechanical stimuli.^[^ ^44^ ^]^ Under magneto‐mechanical stimulation, the YAP translocation in NPSCs was significantly promoted in the Mag‐gel@MF group, whereas the YAP in NPSCs in the other groups remained cytosolic. This result was consistent with the YAP activation accompanied by reduced phosphorylation levels, as shown in Figure 4J–L.

### 2.5 Magneto‐Mechanical Stimulation Promoted Precise Differentiation of NPSCs

**Figure 5:**
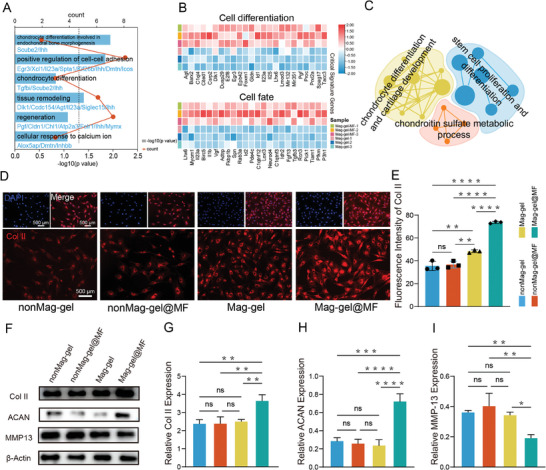
Mechanical stimulation‐driven differentiation of NPSCs. A) GO enrichment analysis of DEG between Mag‐gel@MF and Mag‐gel groups. B) Heatmap of representative DEGs expression levels from GO term associated with differentiation of NPSCs. C) GSEA for DEGs of Mag‐gel@MF‐treated NPSCs. D) Immunofluorescence images of Col II. E) Quantification of fluorescence intensity of Col II in various treatment groups (*n* = 3, **P < 0.01, ****P < 0.0001). F) Detection of Col II, ACAN, MMP‐13, and β‐actin protein levels in nonMag‐gel, nonMag‐gel@MF, Mag‐gel, and Mag‐gel@MF groups using WB. Expression levels of G) Col II, H) ACAN, and I) MMP‐13 protein in NPSCs across various treatment at day 7 post‐treatment (*n* = 3; *P < 0.05, **P < 0.01, ***P < 0.001, and ****P < 0.0001). All statistical data are presented as mean ± SD.

Previous studies have indicated that a frequency of 0.5 Hz can induce the differentiation of stem cells toward the chondrogenic lineage.^[^ ^31^, ^32^ ^]^ After effective cell expansion, we subsequently increase the frequency to 0.5 Hz for MF application. Following magneto‐mechanical stimulation of NPSCs, DEGs in the Mag‐gel@MF group were primarily enriched in biological processes related to chondrocyte differentiation, regeneration, tissue remodeling, and cellular response to calcium ions, compared to Mag‐gel group(**Figure** **5**A,B). The GSEA revealed significant enrichment of biological processes such as stem cell proliferation and differentiation, chondrocyte differentiation, cartilage development, and chondroitin sulfate metabolic processes following treatment with Mag‐gel@MF (Figure 5C). These results suggested that magneto‐mechanical stimulation synergistically promoted NP‐like differentiation of NPSCs. Therefore, we next eamined the effects of magneto‐mechanical stimulation on NPSC differentiation. This study investigated collagen type II (Col II) and aggrecan (ACAN) as NP cell‐specific markers, and detected matrix metalloproteinase 13 (MMP‐13) expression as an NP degeneration‐related marker. The highest Col II expression was observed in the Mag‐gel@MF group (Figure 5D). However, Col II was rarely observed in the nonMag‐gel and nonMag‐gel@MF groups (Figure 5E). In addition, western blot analysis was employed to assess the relative protein expression levels of ACAN, Col II, and MMP‐13. The Mag‐gel@MF group showed the highest NP relative protein expression (Figure 5F–I), whereas no significant relative protein increase was observed in other groups. These results demonstrated that magneto‐mechanical stimulation activated mechanosensitive ion channels, leading to an increased dephosphorylation of the nuclear transcription factor‐YAP. This augmented the expression of genes pertinent to cell proliferation and lineage differentiation, thereby providing a foundation for reversing degenerated IVDs.

### 2.6 PdH Nanozymes Function in Immunomodulation by ROS‐Scavenging and Inflammation Attenuation

**Figure 6:**
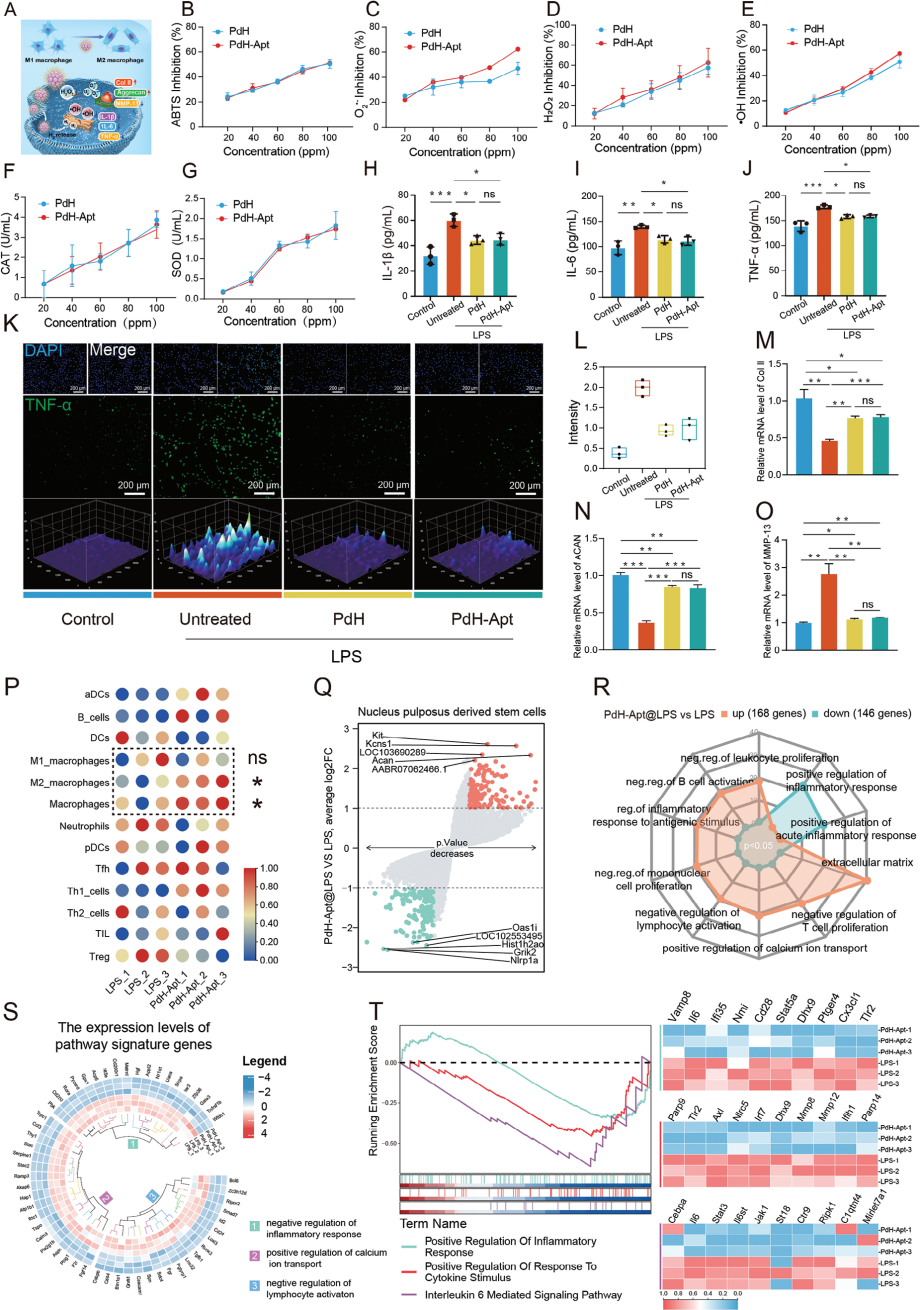
In vitro ROS‐scavenging and anti‐inflammatory properties of PdH and PdH‐Apt. A) Schematic depiction of the multifaceted therapeutic mechanisms of PdH‐Apt on IDD, including ROS‐scavenging, anti‐inflammatory activities, and matrix synthesis activation. B) Evaluation of ABTS (*2,20*‐azinobis) radical scavenging capacity of PdH and PdH‐Apt at varying concentrations (*n* = 3). C) O_2_^•–^‐, D) H_2_O_2_‐, and E) •OH‐scavenging capabilities of PdH and PdH‐Apt (*n* = 3). F) Catalase (CAT)‐ and G) Superoxide dismutase (SOD)‐like activities of PdH and PdH‐Apt (*n* = 3). H) IL‐1β, I) IL‐6, and J) TNF‐α in LPS‐induced NPSCs under different treatments, including control, LPS, LPS + PdH, and LPS + PdH‐Apt (*n* = 3, *P < 0.05, **P < 0.01, ***P < 0.001). K) Immunofluorescent imaging and L) relative fluorescence intensity quantification of TNF‐α in LPS‐induced NPSCs following different treatments. M) Col II, N) ACAN, and O) MMP‐13 mRNA expressions in different treatments (*n* = 3, *P < 0.05, **P < 0.01, ***P < 0.001). P) Single‐sample gene set enrichment analysis (ssGSEA) of differences in infiltration of immune cell types in LPS and LPS + PdH‐Apt treatment groups. Q) DEG volcano map comparing LPS + PdH‐Apt versus LPS treatment. Red dots signify 161 DEGs with substantial upregulation, blue dots indicate 143 DEGs with substantial downregulation, while gray dots represent unchanged genes. R) Radar plot showing GO enrichment of DEGs in LPS + PdH‐Apt versus LPS treatment. S) The expression levels of pathway signature genes. T) GSEA for DEGs of LPS + PdH‐Apt‐treated NPSCs. All statistical data are presented as mean ± SD.

IDD progression is accompanied by a localized cascade of reactive oxygen species (ROS) that intensifies the inflammatory response and creates an unfavorable environment for endogenous regeneration.^[^ ^45^ ^]^ Therefore, we proceeded to evaluate the role of PdH nanozymes in eliminating ROS and inflammatory factors in vitro (**Figure** **6**A). ROS‐scavenging performance of the PdH and PdH‐Apt was evaluated in this study using pical *2,20*‐azinobis (*3*‐ethylbenzthiazoline‐*6*‐sulfonate). Both PdH and PdH‐Apt exhibited significant antioxidant capabilities that correlated positively with their concentrations (Figure 6B). Furthermore, the PdH and PdH‐Apt nanoenzymes showed an equivalent ability to scavenge ROS, as evidenced by the inhibitions of H_2_O_2_, superoxide anion (O_2_^•–^), and •OH (Figure 6C–E). These results indicated that the coupling aptamers exerted no substantial influence on the catalytic efficacy of the PdH nanoenzyme. Moreover, PdH and PdH‐Apt exhibited similar catalase‐like properties, involving the decomposition of H_2_O_2_ to produce O_2_ in a dose‐dependent manner (Figure 6F). As the concentration of PdH‐Apt reached 100 ppm, ≈60% of the H_2_O_2_ was eliminated (Figure 6D). In this study, we observed super‐oxide dismutase‐like properties of the PdH and PdH‐Apt, which eliminated the O_2_^•–^ (Figure 6G), while the PdH‐Apt cleared ≈50% of the O_2_^•–^ radicals at 80 ppm (Figure 6C). The regulatory abilities of the PdH and PdH‐Apt nanoparticles on NPSC responses to inflammation were further investigated using ELISA (Figure 6H–J). After lipopolysaccharide (LPS) treatment, pro‐inflammatory cytokines, specifically interleukin‐6 (IL‐6), interleukin‐1β (IL‐1β), and tumor necrosis factor‐α (TNF‐α), exhibited significant elevation in the NPSCs. However, *co*‐incubation with PdH or PdH‐Apt significantly suppressed the production of the aforementioned pro‐inflammatory cytokines. The TNF‐α level in NPSCs was confirmed to be elevated by LPS through immunofluorescence staining and was dramatically reduced by PdH or PdH‐Apt (Figure 6K,L). These results suggested that both PdH and PdH‐Apt were effective in reducing ROS and inflammation, making them potential candidates for slowing IDD progression.

The dysregulation in ECM synthesis and degradation within the IVD is intricately linked to the local inflammatory milieu.^[^ ^45^ ^]^ This imbalance can trigger and facilitate IDD progression. We investigated the effect of the PdH‐Apt on ECM protein expression in LPS‐treated NPSCs. Col II and ACAN are the primary constituents of the ECM in NP tissues and are responsible for the physiological functions of normal IVD. The qRT‐PCR analysis indicated a reduction in Col II and ACAN expressions in the NPSCs following LPS treatment, a decrease that was notably reversed by both PdH and PdH‐Apt (Figure 6M,N; and Table S3, Supporting Information). Similarly, western blot experiments revealed that nanozyme therapy mitigated the reduced protein synthesis of Col II and ACAN induced by LPS treatment (Figure S14, Supporting Information). Furthermore, the expression of MMP‐13, a gene related to catabolism in the ECM, was significantly downregulated in both the PdH and PdH‐Apt groups (Figure 6O). This suggested that the amelioration of inflammation by PdH‐Apt might orchestrate a balance between anabolism and catabolism in the ECM. A previous study demonstrated that macrophages had the highest level of interaction compared with other cell types, which was a pathological indicator of disc degeneration.^[^ ^46^ ^]^ Thus, we investigated the impact of PdH‐Apt on the macrophage immunological response (Figure S15, Supporting Information). Flow cytometric analysis revealed a significant reduction in the CD86^+^ macrophage population after 24 h of *co*‐incubation with the PdH or PdH‐Apt. In contrast, the PdH and PdH‐Apt groups exhibited a notable augmentation in the CD206^+^ macrophage population. Macrophage polarization was characterized by calculating the percentages of CD206^–^/CD86^+^ M1 and CD206^+^/CD86^−^ M2 macrophages.^[^ ^47^ ^]^ Figure S15 (Supporting Information) shows that both PdH and PdH‐Apt significantly increased the proportions of CD206^+^/CD86^–^ M2 macrophages and decreased the CD206^–^/CD86^+^ M1 macrophage populations. This indicated that PdH and PdH‐Apt successfully reprogrammed inflammatory M1 macrophages toward reparative M2 phenotypes. Furthermore, PdH and PdH‐Apt effectively inhibited LPS‐induced elevations of iNOS and TNF‐α levels, confirming their immunomodulatory role in macrophages (Figure S16, Supporting Information).

To investigate the impact of nanozymes on the inflammatory microenvironment, we analyzed immune cell infiltration in the Mag‐gel@PdH‐Apt@MF treatment and acupuncture groups. The single‐sample gene set enrichment analysis (ssGSEA) results showed an increase in the macrophage populations, with a prominent increase in M2 macrophages, whereas the number of M1 macrophages remained unchanged (Figure 6P). These findings supported the efficiency of nanozyme therapy in promoting macrophage differentiation toward the M2 phenotype. To elucidate the underlying mechanism of the PdH‐Apt nano‐therapy in the LPS‐treated NPSCs, we performed high‐throughput transcriptomic analysis. The volcano plot depicted in Figure 6Q revealed that following PdH‐Apt treatment, 304 genes exhibited significant differential expression, among which 161 were up‐regulated and 143 were down‐regulated. Further GO analysis was conducted to investigate the biological functions of the DEGs. The results suggested that treatment with the PdH‐Apt negatively regulated leukocyte proliferation, inflammatory response, and activations and proliferations of lymphocyte and mononuclear cells. This indicated that the PdH‐Apt had the potential to effectively the inflammatory response (Figure 6R). RNA‐seq analyses revealed that NPSCs treated with the PdH‐Apt showed an enrichment of genes related to the negative regulation of the inflammatory response, positive regulation of calcium ion transport, and negative regulation of lymphocyte activation (Figure 6S; and Table S4, Supporting Information). The GSEA findings revealed significant suppression of key pathways, including those associated with the positive regulation of inflammatory response, positive regulation of response to cytokine stimulus, and interleukin‐6‐mediated signaling pathway following treatment with PdH‐Apt. In contrast, genes involved in positive regulation of the inflammatory response were negatively enriched across the entire transcriptome (Figure 6T). These findings suggested that PdH‐Apt possessed robust ROS scavenging and anti‐inflammatory properties.

### 2.7 Mag‐Gel@PdH‐Apt@MF Ameliorated Disc Degeneration In Vivo

**Figure 7:**
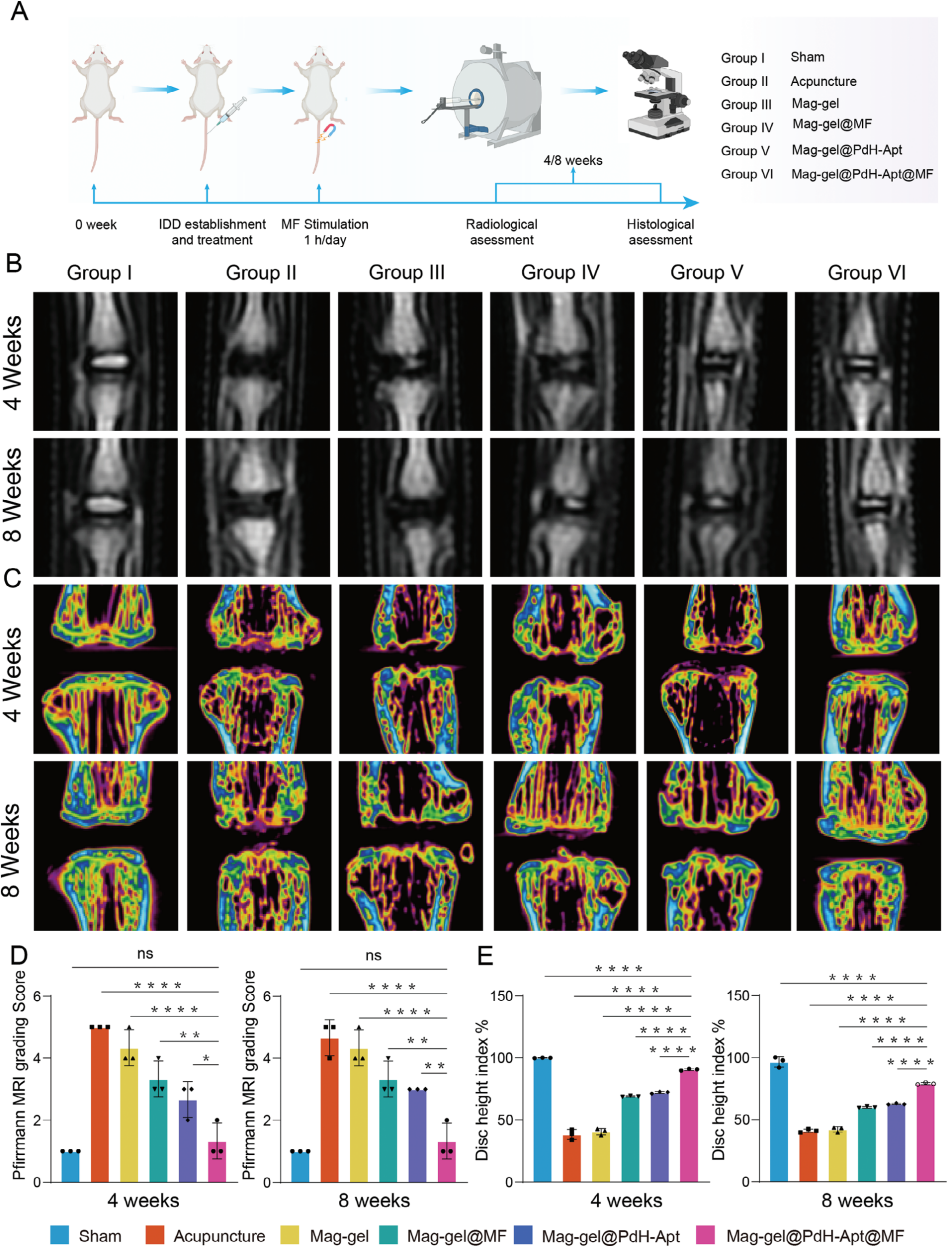
Radiological assessment of animal experiments. A) Schematic diagram of animal experimental procedures. Representative images of each group at 4‐ and 8‐weeks post‐treatment, involving B) MRI and C) micro‐computed tomography (CT) quantitative analyses of D) Pfirrmann grading score and E) height index (% DHI) in each group (*n* = 3; *P < 0.05, **P < 0.01, ***P < 0.001, and ****P < 0.0001). All statistical data are presented as mean ± SD.

A rat IDD model was developed to assess the in vivo therapeutic efficacy of the Mag‐gel@PdH‐Apt system. To minimize additional damage caused by hydrogel administration, the hydrogel was injected into the intervertebral region via the same needle pathway used during the model establishment. Radiological and histological assessments were conducted at 4‐ and 8‐weeks after surgery (**Figure** **7**A). Magnetic resonance imaging (MRI) was employed for noninvasive monitoring of IVD degeneration. The MRI showed that the Mag‐gel@PdH‐Apt@MF group had a higher signal intensity, indicating a higher water content in the NP tissues. Furthermore, animals in the Mag‐gel@PdH‐Apt@MF group showed a significant improvement in the Pfirrmann grading score compared with other groups (Figure 7B,D; and Table S5, Supporting Information). Considering collapse status of the IVD structure was an important factor in grading IVD degeneration, we evaluated the disc height index (DHI) using a micro‐computed tomography (CT) analysis. The study confirmed a successful modeling of disc degeneration, as the DHI decreased over time after needle puncture. At 4‐ and 8‐weeks post‐surgery, the Mag‐gel@PdH‐Apt@MF group had the closest DHI to the control group. These results suggested that the Mag‐gel@PdH‐Apt restored disc height in rats exposed to magnetic stimulation (Figure 7C,E).

**Figure 8:**
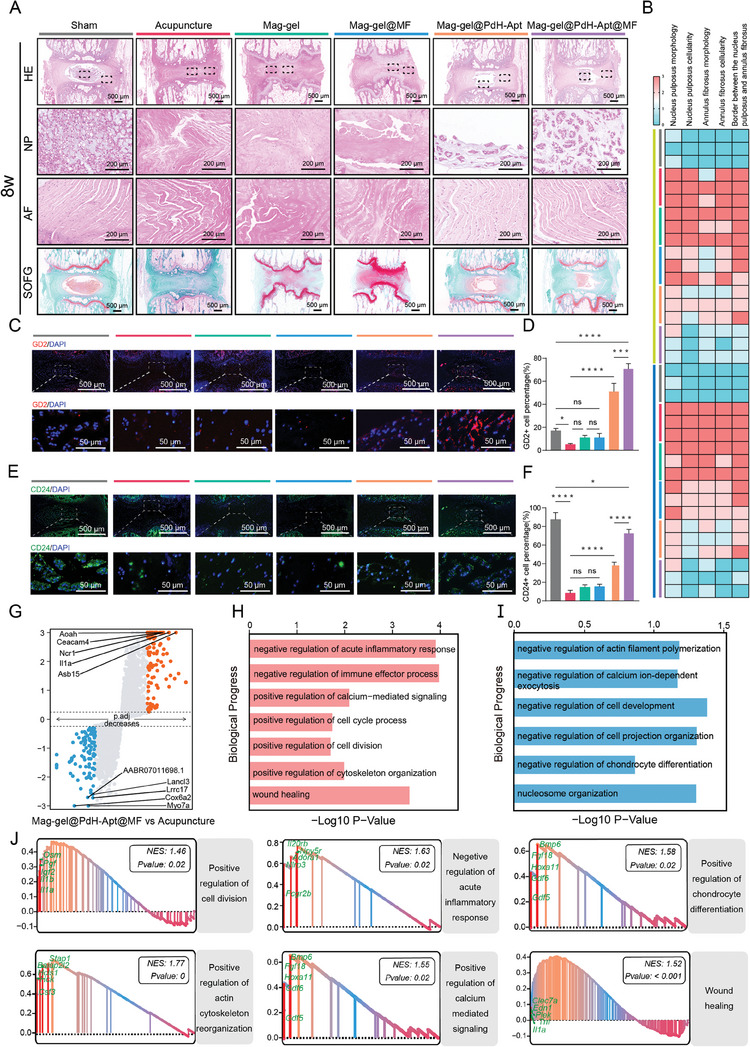
Histological assessment following various in vivo treatments. A) Representative images of Hematoxylin‐eosin (H&E) and Safranin O/fast Green (SOFG) staining of IVD tissues from different groups at 8‐weeks post‐operation. B) Histological grading of IVD from different treatment groups at 4‐ and 8‐ weeks post‐operation. C) Immunofluorescence staining of GD2. D) Quantification of the proportion of GD2^+^ cells (*n* = 3, *P < 0.05; ***P < 0.001; and ****P < 0.0001). E) Immunofluorescence staining of CD24. F) Quantification of the proportion of CD24^+^ cells (*n* = 3, *P < 0.05; ****P < 0.0001). G) DEG volcano map comparing Mag‐gel@PdH‐Apt@MF versus acupuncture treatment. Red dots signify 175 DEGs with substantial upregulation, blue dots indicate 176 DEGs with substantial downregulation, whereas gray dots represent unchanged genes. GO enrichment analysis of H) up‐ and I) down‐regulated DEGs between Mag‐gel@PdH‐Apt@MF and acupuncture treatments. J) GSEA results of DEGs. All statistical data are presented as mean ± SD.

Histological analysis was performed to evaluate the therapeutic efficacy of Mag‐gel@PdH‐Apt in rats with IDD (Table S6, Supporting Information). As depicted in **Figure** **8**A, histological analysis revealed disorganization of the AF and severe disruption of the boundary between the AF and NP in the acupuncture group. Mag‐gel@PdH‐Apt enhanced histological repair compared to the acupuncture group, whereas treatment with Mag‐gel or Mag‐gel@MF did not result in significant histological improvement. Therefore, we deduced that the intrinsic antioxidant properties of magnetic particles did not introduce any additional impact on the experimental outcomes. Furthermore, performing solely mechanical training without stem cell recruitment failed to achieve the desired therapeutic effects, underscoring the critical importance of temporal regulation in treatment. Histological improvement in the IVD was significant in the Mag‐gel@PdH‐Apt@MF group. The disc exhibited a well‐organized structure of AF and NP, with clear borders between them in the Mag‐gel@PdH‐Apt@MF group at 8 weeks postoperatively (Figure 8A; Figure S17, Supporting Information). Safranin O/fast Green (SOFG) staining showed that the Mag‐gel@PdH‐Apt@MF group exhibited elevated levels of proteoglycan compared to those of the other groups at 4‐ and 8‐weeks post‐treatment. Furthermore, the histological scores of IVD in Mag‐gel@PdH‐Apt@MF group were significantly lower than those of the other treatment groups, indicating improvements in NP cells count and morphology, ECM staining, and tissue microstructure (Figure 8B).

We examined the involvement of endogenous NPSCs during disc regeneration by immunostaining for GD2 at 8 weeks postoperatively (Figure 8C,D). A small number of GD2^+^ cells were observed in the groups without DB67 aptamers, such as the Mag‐gel and Mag‐gel@MF groups. In contrast, the abundance of GD2^+^ cells notably increased in the hydrogel modified with DB67, specifically in the Mag‐gel@PdH‐Apt and Mag‐gel@PdH‐Apt@MF groups. This suggested that the modification of the DB67 aptamer attracted native NPSCs to the damaged disc region, while MF does not effectively recruit cells. Intriguingly, magnetic stimulation increased the number of GD2^+^ cells in the Mag‐gel@PdH‐Apt@MF group, indicating that magneto‐mechanical actuation might enhance the in situ proliferation of NPSCs. Since the in vitro experimentsdemonstrated that magneto‐mechanical actuation could promote the differentiation of NPSCs into terminated NP‐like cells, we delved deeper into examining the expression of CD24, a specific NP cell marker, in the IVD (Figure 8E,F). Within the acupuncture group, there was a significant reduction in the population of CD24^+^ cells, which concurrently exhibited a loss of their intrinsic phenotype. The Mag‐gel@PdH‐Apt group showed a significant increase in CD24^+^ cells, possibly because of the recruitment of NPSCs and their spontaneous differentiation into NP‐like cells. The number of CD24^+^ cells further increased in rats after treated with the Mag‐gel@PdH‐Apt@MF, indicating that magneto‐mechanical actuation facilitated the differentiation of NPSCs into NP‐like cells in vivo. Subsequently, we utilized RNA‐seq to conduct a comprehensive analysis of the DEGs in the NP tissues of the treatment and acupuncture groups, aiming to uncover the molecular mechanisms and pathways mediated by the functionalized system on therapy and regeneration. A volcano plot (|log_2_ FC| >1 and *P <* 0.05) showed that 175 genes were upregulated and 176 were downregulated in NP tissues treated with the Mag‐gel@PdH‐Apt@MF (Figure 8G). GO analysis revealed that the up‐regulated DEGs were primarily linked to cell division, calcium‐mediated signaling, negative regulation of the inflammatory response, and positive regulations of both cytoskeleton organization and cell cycle processes (Figure 8H,I). GSEA showed that the treatment group had a significant increase in “positive regulation of cell division,”“negative regulation of acute inflammatory response,”“positive regulation of chondrocyte differentiation,” “positive regulation of actin‐cytoskeleton reorganization,” “positive regulation of calcium‐mediated signaling,” and “wound healing” (Figure 8J). These results demonstrated that the Mag‐gel@PdH‐Apt@MF approach recruited NPSCs and promoted their proliferation and differentiation in vivo.

## 3 Discussion

IVDD is characterized by the loss of proteoglycans and the disorganization of the ECM architecture.^[^ ^48^ ^]^ This is attributed to a vicious circle of three intertwined conditions: cellular exhaustion, reduced cell viability, and inflammation or catabolic cascades.^[^ ^49^ ^]^ Strategies including the delivery of anti‐inflammatory cytokines, growth factors, or exogenous cells, that aim to correct a single pathological process have shown promise.^[^ ^50^, ^51^ ^]^ However, they cannot generate sufficient momentum to support complete IVD restoration. To address this challenge, we proposed a synthetic strategy that was controllable remotely and integrated physical and chemical cues to overcome the major pathological processes of IVD to orchestrate disc regeneration.

Early indicators of IDD include reduced cell numbers and phenotypic alterations in resident NP cell populations.^[^ ^52^ ^]^ Therefore, cell supplementations of several promising candidates, such as NP cells, notochordal cells, chondrocytes, and mesenchymal stem cells (MSCs), have been investigated extensively in preclinical and clinical researches.^[^ ^53^, ^54^ ^]^ However, the harsh local microenvironment of the IVD, characterized by conditions of hypoxia and nutrient scarcity, coupled with metabolic disturbances and intense mechanical pressures, presents significant obstacles for the survival of transplanted cells.^[^ ^55^ ^]^ Recently, scientists have discovered a population of endogenous stem cells within the IVD that have a great potential to regenerate NP tissues naturally due to their innate adaptation to the local microenvironment.^[^ ^16^ ^]^ The presence of Tie2^+^/GD2^+^ NPSCs in IVD tissues was further validated by immunohistochemical staining and single‐cell database analysis. These findings highlight the potential of regenerative cells for IDD repair.

To achieve therapeutic success, it is crucial to guide the migration of endogenous stem cells to the location of the damage. Numerous methods have been suggested to regulate stem cell recruitment, including cell‐adhesive antibodies, growth factors, and ECM‐based biomaterials.^[^ ^56^, ^57^ ^]^ Recently, nucleic acid aptamers have gained attention because of their small physical size, customizable structure, easy chemical modification, low immunogenicity, and high stability.^[^ ^58^, ^59^ ^]^ The nucleic acid aptamer Apt19s has been investigated for its ability to augment the repair of bone and cartilage by selectively binding to and recruiting BMSCs.^[^ ^58^, ^60^ ^]^ In this study, aptamer DB67 was used to target GD2, resulting in robust and selective binding that facilitated the recruitment of NPSCs from the outer to inner zones to facilitate IVD regeneration.

Tissue regeneration depends on the availability of a sufficient number of stem cells. Our analysis revealed that GD2^+^/Tie2^+^ positive stem cells accounted for only 1% of IVD cells. These cells progressively decline during aging, exacerbating the shortage of NPSCs and limiting their regenerative capacity. Conventional approaches of functionally modulating cell proliferation through chemical stimulations in a binary on/off manner have clear limitations, as they disrupt normal cellular functioning.^[^ ^61^ ^]^ In contrast, the subsequent iteration of the remote stimulation method utilizes physical cues to provide precise control over biological targets. A novel technique for expanding MSCs has been developed by subjecting MSCs to magnetically‐driven mechanical stimulation in a 3D environment. Dynamic mechanical stimulation regulated MSC development and proliferation in that experiment by enhancing the interaction between the matrix and integrin β1 via the FAK‐ERK pathway.^[^ ^30^ ^]^ Moreover, magnetism has exhibited exceptional characteristics, such as adjustable magnitude, noninvasive in vivo manipulation, and precise temporal on/off control, making it an ideal choice for biomedical purposes.^[^ ^62^ ^]^

This study introduced a new superparamagnetic nanocomposite hydrogel (Mag‐gel) by incorporating SPIONs into a covalent and hydrogen‐bond‐cross‐linked PAAm/HA hydrogel matrix. An excellent superparamagnetism of the Mag‐gel enabled quick magnetization, which subsequently coupled mechanical force with dynamic magnetic stimulation, providing an accurate external trigger for the fate regulation of NPSCs. Our findings confirmed that magneto‐mechanical stimulation effectively increased the number of NPSCs and boosted their proliferation, both in vitro and in vivo. Mechano‐sensors located on the plasma membrane were activated by mechanical stimuli, which then converted mechanical cues into biochemical signals that regulated cell behavior.^[^ ^63^ ^]^ Prior investigations have revealed that YAP is a master regulator of mechano‐transduction that functions in cell growth and cell cycle re‐entry into the G1 phase.^[^ ^64^ ^]^ Our study found that magneto‐mechanical actuation induced cell cytoskeletal protein remodeling by activating YAP and facilitating its nuclear‐cytoplasmic translocation. Furthermore, we observed unique Ca^2+^ oscillations with robust repetitive Ca^2+^ spikes in NPSCs in response to constant magneto‐mechanical forces. Ca^2+^ is a crucial secondary messenger that serves as a critical factor in the dynamic regulation of diverse cellular processes, including cell proliferation.

One obstacle to disc regeneration is the stimulation of NPSCs to differentiate into cells that can secrete functional ECM to support the regenerative process.^[^ ^4^ ^]^ Various approaches have been proposed to address this issue, including the use of inducible biochemical factors such as BMP‐2 and TGF‐β3, as well as gene‐engineering techniques.^[^ ^65^, ^66^ ^]^ However, achieving a local balance between effectiveness and cytotoxicity in vivo remains challenging. Mechanical force is an effective strategy for manipulating the stem cell differentiation. Furthermore, the IVD is a load‐bearing tissue, and moderate physical exercise is beneficial for functional maintenance. Recently, magnetic nanoparticles tethered to an Arg‐Gly‐Asp peptide were demonstrated to modulate the adhesion and differentiation of stem cells by utilizing dynamic magnetic fields.^[^ ^67^ ^]^ Our study used magnetic‐driven mechanical cues to provide a noninvasive‐training program for NPSCs. This allowed for precise adjustments and resulted in significant upregulations of NP‐specific markers (Col II and ACAN) after magneto‐mechanical training. Moreover, the catabolic marker MMP‐13 was present at low levels at the initial stages of cellular differentiation, and the proportion of CD24^+^ cells was increased by mechanical stimulation. The data indicated that the Mag‐gel was functionalized as a physical training platform, and that the associated mechanical cues effectively directed the recruited NPSCs to differentiate into NP‐like cells, enhancing the synthesis of ECM and providing a strong basis for improving IDD.

NP are avascular and aneural tissues that exhibit immune‐privileged characteristics.^[^ ^20^ ^]^ Therefore, damage to NP tissues can trigger autoimmune responses and downstream cascades, resulting in high ROS levels and inflammation. This creates a complex inflammatory environment that accelerates IDD progression. The harsh local environment of IDD is hostile to cell survival, including that of the recruited NPSCs. Therefore, it is crucial to remove locally‐accumulated ROS to promote endogenous regeneration. Nanozymes, such as cerium dioxide (CeO_2_), trimanganese tetraoxide (Mn_3_O_4_), and vanadium pentoxide (V_2_O_5_), are nano‐catalysts that display catalytic activity and reaction kinetics similar to those of natural enzymes. These compounds are characterized by their capacity to eliminate excessive ROS by mimicking their antioxidant activity.^[^ ^68^, ^69^, ^70^ ^]^ This compound shields cells from damage caused by oxidative stress, making it useful in the fields such as neuro‐protection, cyto‐protection, anti‐inflammation, and cancer therapy.^[^ ^71^ ^]^ Recently, there has been growing interest in novel Pd‐based nanozymes owing to their exceptional biocompatibility and potent antioxidant activity. Within the present research, PdH served as a hydrogen carrier and self‐catalyst nanozyme, effectively scavenging ROS and exerting anti‐inflammatory effects.^[^ ^24^ ^]^ During pathological disc degeneration, NP cells established extensive crosstalk with macrophages, which were recognized as the main effectors regulating the inflammatory response in IDD.^[^ ^46^ ^]^ This study revealed that the PdH‐Apt nanozyme significantly increased the proportions of CD206^+^/CD86^–^ M2 macrophages. These findings suggested that PdH‐Apt effectively reprogrammed macrophages into reparative M2 phenotypes, so as to improve the inflammatory environment and create a favorable environment for endogenous NP regeneration.

In summary, we have presented a straightforward and promising method that combined chemical and physical cues to coordinate IVD regeneration. This pro‐regenerative approach is not restricted to rescuing IDD, as it might also be applied to other degenerative diseases such as osteoarthritis and meniscus injury. Although we have confirmed that triggering the nuclear entry of YAP via mechanical stimulation is an effective method for training stem cells, further exploration is required to understand the mechanism by which downstream genes are regulated. Moreover, investigations in larger animals, including goats, cattle, and rhesus monkeys, are warranted to explore their clinical therapeutic significance.

## 4 Conclusion

This study presents a novel superparamagnetic hydrogel designed for cellular mechanical training with integrated endogenous stem cell recruitment and immune regulatory capabilities to rescue IDD. Endogenous stem cells are recruited using aptamers that specifically bind to NPSCs to reverse the degeneration process. The activated mechanosensitive Ca^2+^ ion channels and YAP translocation via coupled mechanical cues resulted in increased proliferation and the directed differentiation of recruited NPSCs. Furthermore, PdH modification provided an anti‐inflammatory microenvironment, optimizing the healing outcome of IDD. Overall, our research presents a noninvasive and remote chronology synthetic strategy that integrates physical and chemical cues to regulate stem cell fate and accelerate endogenous regeneration of the IVD.

## 5 Experimental Section

### Synthesis of Pd Nanoparticles

Potassium bromide, poly(vinyl pyrrolidone), *L*‐ascorbic acid, and sodium tetrachloropalladate (Na_2_PdCl_4_) were dissolved in *N, N*‐dimethylformamide following by the addition of deionized water and thorough mixing. After standing at room temperature (25 °C) for 24 h, the solution was transferred into a container and heated in a 100 °C oil bath for 3 h. After natural cooling, the Pd nanoparticles were purified and harvested using an Amicon ultrafiltration tube (Millipore, Burlington, MA) and subsequently dispersed in deionized water, shielded from light.

### Synthesis of PdH

Ten milligrams of Pd nanoparticles was dispersed in 10.0 mL of deionized water and transferred to a small sealed bottle. In another small sealed bottle, 200.0 mg of NaBH_4_ was loaded and connected to the first bottle with a rubber tube. Phosphate‐buffered saline was injected into the bottle containing NaBH_4_ to generate hydrogen gas. After a 30 min reaction, PdH was obtained. The sealed bottle was stored in dark.

### Synthesis of PdH‐Apt

The PdH was dissolved in 15.0 mL of ultrapure water by mixing it with 0.1 g of *N*‐acetyl‐*L*‐cysteine (NAC) at a ratio of 1:4 ratio. It was sonicated for 10 min, magnetically stirred for 5 h, and then centrifuged at high speed to obtain the PdH‐NAC. Five microliters of aptamer (Table S1, Supporting Information) was added, and the mixture solution was sonicated and thoroughly stirred for 5 h in an ice bath. Thereafter, it was washed thrice with ultrapure water, followed by centrifugation to collect the precipitate. Subsequently, the samples underwent freeze‐drying and were stored at low temperatures.

### Fabrication of PEG/PEI‐SPIONs

Initially, 30.0 g of polyethylene glycol (PEG) and 0.6 g of polyetherimide (PEI) were heated to 100 °C in a three‐neck flask filled with argon, and 1.4 g of Fe(acac)_3_ powder was added with stirring for 15 min. The temperature was then raised to 280 °C and the reaction was continued for 60 min. As the reaction was cooled naturally at room temperature, the resulting PEG/PEI‐SPIONs were collected and washed thrice in turn with toluene and acetone, and vacuum dried finally.

### Fabrication of Mag‐gel@PdH‐Apt

In brief, 1.2 g of hyaluronic acid sodium and 2.8 g of acrylamide were added into 40.0 mL of deionized water and stirred for 48 h until each component was completely dissolved to form an aqueous solution. During the stirring process, 0.4 g of PEG/PEI‐SPIONs, 1.6 mg of PdH‐Apt, 0.6 g of *1*‐ethyl‐*3*‐(*3*‐dimethylaminopropyl) carbodiimide hydrochloride, 0.6 g of *2*‐morpholinoethanesulfonic acid, 0.6 g of N‐hydroxy succinimide, 4.8 mg of MBAA, 0.021 g of TEMED, and 0.014 g of APS were sequentially added to the solution. Finally, the obtained Mag‐gel@PdH‐Apt solution was degassed in vacuum, swiftly transferred into a 24‐well plate, and the Mag‐gel@PdH‐Apt was obtained within 10 min.

### Characterization of Morphology

The hydrogels were freeze‐dried for 48 h and then cut through the middle to obtain cross sections. A 10 nm layer of gold was sputtered onto the surface of these sections. Subsequently, the surface morphology and microstructure were observed using a scanning electron microscopy (SU8010, HITACHI, Japan) at various magnifications using an acceleration voltage of 10 kV. The average pore size of the hydrogel was analyzed using the Nano Measure software (Nano Measure 1.2, Fudan University, Shanghai, China).

The absorption spectra of Pd and PdH nanoparticles were acquired using a UV–vis–NIR spectrophotometer (UV‐3600 Shimadzu Corporation, Kyoto, Japan) over the wavelength range of 175 to 3300 nm. Following a 48‐h freeze‐drying period, FTIR (Nocolet IS50,Thermo FisherScientific, Waltham, MA) spectroscopy was conducted on the hydrogels to capture their infrared spectra within the range of 500–4000 cm^−1^, with a spectral resolution of 2 cm^−1^. Using an accelerating voltage of 200 kV, transmission electron microscopy (H‐7650, HITACHI, Japan) was employed to capture the morphology of the nanoparticles. X‐ray diffraction (X'Pert PRO MPD, Malvern Panalytical) and X‐ray photoelectron spectroscopy (Thermo Kalpha, Thermo Scientific) were utilized to analyze the chemical composition and crystal structure of the nanoparticles, respectively. Dynamic light scattering (Malvern Zetasizer Nano ZS90, Malvern Panalytical) was employed to determine the zeta potential of the nanoparticles.

### Extraction, Identification, and Culture of NPSCs

Primary NPSCs were isolated following an established protocol. Initially, the NP tissues were extracted from the lumbar discs of 6–8‐week‐old Sprague‐Dawley (SD) rats. Two milliliters of type II collagenase (C8150, Solarbio, Beijing, China) was added to the tissues. After digestion for 15–20 min at 37 °C, twice the volume of cell culture medium containing serum was added to terminate the digestion. After centrifugation, the supernatant was discarded, and the remaining cells and tissue were added with culture medium (containing DMEM/F12 medium; 10% fetal bovine serum; 1% penicillin‐streptomycin), and then transferred to culture dishes after fully suspending. The cells were cultured in an incubator with 5% CO_2_ at 37 °C, and the culture medium was replaced every 3 days until the cell colonies (cell clusters with a diameter >1 mm) were observed. The cell clusters were then separated and collected using a cloning ring for passage culture. Flow cytometry was used to identify and screen the cell population.

### Animals and Surgical Procedures

All animal procedures were approved by the Ethical Committee for Animal Experiments of the Fourth Military Medical University (License No. IACUC‐20241252). In this study, 6–8‐week‐old adult male Sprague‐Dawley rats were used. Anesthesia was induced by 1% solution of sodium pentobarbital administered intraperitoneally. Thereafter, a 20G needle was carefully inserted vertically into the center of the IVD between the 6th and 7th caudal vertebrae, and the needle was rotated 360° before being held at this spot for 30 s. Subsequently, 50 µL of hydrogel was injected into the disc. The experimental design comprised six groups: 1) the sham group; 2) animals treated with acupuncture without subsequent therapy; 3) animals treated with Mag‐gel following acupuncture; 4) animals treated with Mag‐gel@MF after acupuncture; 5) animals treated with Mag‐gel@PdH‐Apt post‐acupuncture; and 6) animals treated with Mag‐gel@PdH‐Apt@MF following acupuncture. After disinfecting the surgical sites, the rats were returned to cages for routine feeding. When applying magnetic stimulation, place the rats in an acrylic chamber measuring 15 × 5 × 5 cm. A magnetic stimulation device was placed beneath the chamber to provide continuous stimulation. Due to spatial constraints, the rats experience minimal movement within the chamber, ensuring consistent stimulation of the target area by the magnetic device. Imaging and histological assessments were conducted at 4‐ and 8‐weeks post‐surgery.

### Statistical Analysis

Statistical analyses were performed using GraphPad Prism 8.0 software (GraphPad Software, Inc., USA). Data were presented as mean ± SD. Statistical tests utilized were the independent samples t‐test and one‐way analysis of variance. Differences were considered statistically significant at *P <* 0.05.

## Conflict of Interest

The authors declare no conflict of interest.

## Supporting information



Supporting Information

Supplemental Video 1

Supplemental Video 2

## Data Availability

The data that support the findings of this study are available from the corresponding author upon reasonable request.
