# Supplementary material for: A Novel Superparamagnetic‐Responsive Hydrogel Facilitates Disc Regeneration by Orchestrating Cell Recruitment, Proliferation, and Differentiation within Hostile Inflammatory Niche
Source: Adv Sci (Weinh). 2024 Oct 7;11(44):2408093. doi: 10.1002/advs.202408093 (PMC11600201; doi:10.1002/advs.202408093)
Supplement: Supplementary file 1 — Supporting Information [file ADVS-11-2408093-s001.docx]

Supporting Information

A Novel Superparamagnetic-Responsive Hydrogel Facilitates Disc Regeneration by Orchestrating Cell Recruitment, Proliferation, and Differentiation within Hostile Inflammatory Niche

Borui Xue, Yan Peng, Yongfeng Zhang, Shijie Yang, Yi Zheng, Huiling Hu, Xueli Gao, Beibei Yu, Xue Gao, Shengyou Li, Haining Wu, Teng Ma, Yiming Hao, Yitao Wei, Lingli Guo, Yujie Yang, Zhenguo Wang, Tingfeng Xue, and Jin Zhang*, Beier Luo*, Bing Xia*, Jinghui Huang*

B. Xue, Y. Zheng, X. Gao, S. Li, H. Wu, Teng. M, Y. Hao, Y. Wei, L. Guo, Y. Yang, Z. Wang, Dr. B. Xia, Prof. J. Huang

Department of Orthopaedics, Xijing Hospital, The Fourth Military Medical University, Xi’an 710032, P.R. China.

E-mail: xiabing8807@fmmu.edu.cn/ huangjh@fmmu.edu.cn

Prof. B. Luo

Department of Spinal Surgery, Shanghai Changhai Hospital, Affiliated to Naval Medical University, Shanghai, 200433, P.R. China.

E-mail: bear_luo@126.com

Prof. J. Zhang

College of Chemical Engineering, Fuzhou University, Xueyuan Road, Fuzhou 350108, P.R. China.

E-mail: J_Zhang929@fzu.edu.cn

Y. Peng

College of Advanced Manufacturing, Fuzhou University, Jinjiang 362200, P.R. China

B. Xue, H. Hu

Air Force 986(th) Hospital, The Fourth Military Medical University, Xi'an, 710032, P.R. China.

Y. Zhang, S. Yang, B. Yu

Department of Neurosurgery, The Second Affiliated Hospital of Xi'an Jiao Tong University.

Xi'an 710032, P.R. China.

X. Gao, T. Xue

School of Ecology and Environment, Northwestern Polytechnical University, Xi’an 710072, P.R. China.

**Supplementary Experimental Section:**

*Swelling behavior:* Swelling ratio of the hydrogels was calculated from the mass change of the samples (*ca.* 5.0 × 5.0 × 4.0 mm^3^) soaked in deionized water for 72 h. Surface water was carefully removed before weighing. The swelling ratios of the hydrogels were calculated using formula (1):

Swelling ratio (%)＝ $\frac{W_{t}}{W_{0}}$×100% (1)

, where *W*_t_ represented the weight of the swollen hydrogel after being immersed in water for a period of time and *W*_0_ represented the initial weight of the hydrogel.

*In Vitro Degradation Properties:* The hydrogels were placed in a PBS (10.0 mL) containing 3.0 mg α-chymotrypsin to simulate the in vivo microenvironment. All samples were placed in a 37 °C constant-temperature shaker at a speed of 100 rpm. At different time intervals, the hydrogels were dried and weighed to evaluate degradation behavior. The degradation properties of hydrogels were assessed using formula (2):

Weighting remaining (%)＝$\frac{W_{t}}{W_{0}}$×100% (2)

, where *W*_0_ represented the weight of the initial hydrogel and *W*_t_ represented the weight of the hydrogel after culturing for a desired period.

*Mechanical measurement:* Hydrogel disks (10-mm width, 30-mm length, and 2-mm thickness) were prepared and then secured at both ends using metal clamps onto a universal material testing (UMT; MTS/SANS) machine. Subsequently, a tensile test was conducted at a speed of 50.0 mm·min^–1^ within a ambient temperature of 25 °C until the hydrogel fractured and stopped stretching. Following the experiment, stress-strain curves were generated, and the tensile modulus and tensile strength of the hydrogels were calculated.

Cylindrical hydrogels (diameter:15.0 mm, height:10.0 mm) were fabricated and positioned between two parallel plates, after which compression tests were conducted on hydrogels using a 10 N loading unit from a UMT at ambient temperature. The samples were applied with an 80% strain at a loading rate of 5 mm·min^−1^.

The recovery of the hydrogels was evaluated using UMT, and the tensile and compression conditions were as described above. By contrast, the strains in the tensile and compressive tests were 300% and 60%, respectively. Ten repetitions of the loading and unloading were performed. Each group underwent three measurements, and the averages were calculated.

*Vibrating sample magnetometer (VSM) analysis*: The magnetic properties of the nanoparticles and freeze-dried hydrogels were assessed using a VSM (Lake Shore 7404, USA) at room temperature with an applied field up to 20 kG.

*Device and calculation of external magneto-induced dynamic mechanical stimulation:* To achieve dynamic magneto-mechanical stimulation on the Mag-gel@PdH-Apt hydrogel, a neodymium permanent magnet (400 mT) was installed on a reciprocating actuator. The hydrogel constructs were attached to the bottom of a well plate or Petri dish, and cyclic magnetic forces were transduced into mechanical perturbations on the loaded cells. Because the magnetic field strength (or magnetic flux density) of the permanent magnet was inversely proportional to the distance, the force amplitude of each TMP (FTMP) was calculated as:

F_mag_＝$\frac{V_{p}\times{\Delta X}_{p}}{\mu_{0}}\left( B\cdot\nabla\right)B$ (3)

, where *V*_p_ was the average volume of the magnetic microparticles, *ΔX*_p_ was the magnetic susceptibility, *µ_0_* was the magnetic permeability, *Β·∇* was the experimentally determined relationship between the magnetic field and distance to the permanent magnet, and *B* was the applied magnetic field. In this experiment, the stimulation duration was 2 h/day for 7 days. Based on previous research,^[28-30]^ we applied MF to cells at a frequency of 0.1 Hz to promote proliferation and at 0.5 Hz to induce differentiation. The range of the applied force was adjusted by changing the distance between the hydrogel and magnet.

*Fluorescent ubiquitination-based cell cycle indicator (FUCCI) system:*A FUCCI reporter gene (EFLA-mKO2-T2A-mAzami-Green) overexpression lentiviral vector (Genechem, China) was constructed, which specifically expresses in certain stages of the cell cycle. By utilizing reporter genes associated with different fluorescent colors, it enables real-time imaging of the different phases of the cell cycle. The red, yellow, and green fluorescence corresponds to cells in the G1, G1/S transition, and S/G2-M phases, respectively. According to the manual, the lentiviral vector was used to infect and label NPSCs. Subsequently, the labeled NPSCs were subjected to different treatments, and observed and statistically analyzed using ImageXpress Micro Confocal (Molecular Devices).

*Processing and analysis of single-cell RNA sequencing data:* In single-cell genomics, the processed data were analyzed with the ‘Seurat’ package using the Seurat (v4.3.2) R toolkit. Cells with less than 500 expressed genes, mitochondrial gene proportions exceeding 15% were excluded. High-variance genes were identified using the ‘Find Variable Genes’ function, followed by principal component analysis. Uniform Manifold Approximation and Projection (UMAP) was utilized for dimensionality reduction and visualization. Differential expression analysis was performed using the ‘Find All Markers’ function with thresholds of | log2(fold-change) | > 0.3 and adjusted p < 0.05.

*Bulk RNA sequencing and bioinformatics analysis:* TRIzol reagent was used to extract total RNA. Total RNA was isolated and RNA-Seq libraries were prepared using the NEBNext Ultra™ Illumina RNA Library Prep Kit through paired-end sequencing. Raw data were quality controled to generate “clean reads” for further analysis. The differentially expressed genes (P value ＜ 0.05, |Fold Change| ≥ 1) were subjected to the enrichment analysis of functional enrichment analysis. For immune infiltration analysis, the degree of immune cell infiltration in each sample was calculated using the ssGSEA algorithm. After downloading the gene set data in “gmt” format with 13 immune-related score, the “GSVA” package was used to score each sample in corresponding dataset. Subsequently, the differences in the expression of 13 immune cells were further distinguished.

*Live/Dead assay:*A Live/Dead cell imaging kit (Invitrogen) was employed to conduct live/dead cell experiments. Live cells were stained with green fluorescence, whereas dead cells were labeled with red fluorescence. Initially, NPSCs were mixed with hydrogels and cultured for 72 h. Subsequently, the cultures were washed three times with phosphate-buffered saline (PBS). After that, the staining solution was added and the samples were incubated at 37 °C for 30 min. Finally, the results were observed and recorded using a confocal microscope (Olympus, FV3000).

*Gene expression analysis through quantitative real-time polymerase chain reaction (qRT-PCR):* Total RNA was extracted from NPSCs using an RNA extraction kit (QIAGEN, Germany). Subsequently, the extracted RNA was subjected to a reverse transcription reaction using the PrimeScript^TM^ RT Master kit (TAKARA, China), resulting in the generation of complementary DNA (cDNA). Finally, the relative expression levels of the target gene’s mRNA were quantitatively analyzed using the CFX96 PCR system (Bio-Rad, Australia). The specific primer sequences are detailed in Table S2 (Supporting Information).

*Immunofluorescence:* NPSCs were washed three times with PBS before being fixed in 4% paraformaldehyde for 20 min. the cells were then permeabilized with 0.2% Triton X-100, and finally blocked with 10% goat serum for 1 h. The cells were then incubated with the primary antibody at 4 °C overnight, after which they were incubated with a fluorescently labeled secondary antibody at room temperature for 1 h. Upon completion of the incubation, the cells were stained with DAPI for nuclear visualization. Finally, the cellular morphology and fluorescence staining were observed using a confocal microscope (Olympus, FV3000), and parameters such as fluorescence intensity and cell area were quantitatively analyzed using ImageJ software (NIH, USA).

*Western blotting analysis:* The treated cells were washed three times with PBS, followed by extraction of total cellular proteins using RIPA buffer. The concentration was determined using a BCA protein assay kit (Beyotime). Subsequently, the extracted total proteins were separated by SDS-PAGE gel (Beyotime), and then transferred onto a polyvinylidene fluoride (PVDF) membrane. The membrane was blocked with 5% skim milk at room temperature for 1 h, followed by overnight incubation with primary antibody at 4 °C, Then the membrane was incubated with secondary antibody for 2 h. Finally, protein bands were visualized and analyzed using a chemiluminescence detection system (GE Healthcare).

*Transwell experiments:* To assess the impact of aptamers on NPSCs recruitment, hydrogels containing or not containing aptamers were placed in the bottom chamber of Transwell (NUCN), while NPSCs were seeded in the top chamber. After adding culture medium, the cells were allowed to incubate for 24 h, followed by careful removal of the cells on the surface of the top chamber. Subsequently, the cells were fixed with 4% paraformaldehyde and stained with crystal violet. Finally, under microscopic examination, the migrated cells were counted and analyzed using ImageJ software (NIH, USA).

*Migration of NPSC co-cultured with hydrogels:* Two distinct types of hydrogels, one with and another without the DB67 aptamer, were fabricated, and NPSCs were seeded on the surface of these hydrogels. After 1, 3, and 7 days of culture, Live/Dead staining was conducted. Subsequently, confocal microscope (Olympus, FV3000) was employed to perform serial section scanning of the hydrogels in order to monitor the quantity and depth of cell migration. Sections at various depths were selected, and the number of cells in each group was quantitatively analyzed using ImageJ software (NIH, USA).

*Calcium imaging:* A calcium ion detection kit (Fluo-8, AM) was utilized to visualize intracellular calcium ions signals in live cells. The hydrogels containing NPSC were washed three times with calcium-free PBS. Following this, the staining working solution (2 μM) was added, and the hydrogels were incubated at 37 °C in cell culture incubator for 1 h. Imaging was then recorded using a fully automated intelligent imaging system (Thermo Fisher EVOS M7000). Throughout the recording period, the hydrogel was stimulated with a permanent magnet for 10 seconds, with 1-minute interval between each stimulation.

*ROS-scavenging capacities of PdH and PdH-Apt:* The antioxidant capacities of PdH and PdH-Apt was assessed using the ABTS free radical scavenging assay kit (Solarbio). Following the instructions, different concentrations of PdH and PdH-Apt (20, 40, 60, 80, and 100 ppm) were thoroughly mixed with the working solution and allowed to stand at room temperature under light avoidance for 6 min. Subsequently, the supernatant was collected by centrifugation, and the absorbance at 405 nm was measured using the microplate reader (Bio Tek).

*H_2_O_2_-scavenging assays of PdH and PdH-Apt:* To evaluate the H_2_O_2_ scavenging capabilities of PdH and PdH-Apt, nanoparticles of different concentrations (20, 40, 60, 80, and 100 ppm) were incubated with a solution of 1.0 mM H_2_O_2_ for 1 h. Following this, the concentration of H_2_O_2_ was determined using a H_2_O_2_ detection kit (Solarbio), with the protocol provided in the kit manual. Absorbance was measured at a wavelength of 415 nm using the microplate reader (Bio Tek).

*CAT-like activity assays of PdH and PdH-Apt:* To assess the catalase (CAT) activity of PdH and PdH-Apt, a CAT assay kit (Solarbio) was employed to detect the CAT activity of nanoparticles at various concentrations ((20, 40, 60, 80, and 100 ppm)) following the instructions in the manual. The absorbance at 240 nm was recorded using the microplate reader (Bio Tek).

*SOD-like activity and O_2_^•−^ scavenging assays of PdH and PdH-Apt:* The Superoxide Dismutase (SOD) activity of PdH and PdH-Apt at different concentrations was evaluated using an SOD activity assay kit. The experiment weas conducted according to the instructions, the absorbance at 450 nm was measured usin the microplate reader (Bio Tek).

*ELISA:* NPSCs were seeded into a 24-well plate and allowed to adhere. The control group was cultured with DMEM/F12, whereas the un-treatment group was treated with DMEM/F12 containing 100ng/L lipopolysaccharide (LPS). For the remaining experimental groups were respectively treated with PdH and PdH-Apt for 2 h. Following this, all groups were further cultured with DMEM/F12 containing LPS. Subsequently, the expression levels of tumor necrosis factor-α (TNF-α), interleukin-1β (IL-1β), and interleukin-6 (IL-6) in the NPSCs from each group were quantified using an enzyme-linked immunosorbent assay (ELISA) kit (Bioswamp).

*Radiological examination:* At 4- and 8-weeks post-operation, micro-CT scans of the vertebral disc were conducted using the GE eXplore Locus SP system (Fairfield, USA) with settings of 14-mm resolution, 27-mm voxel size, threshold of 1150, tube voltage of 80 kV, current of 80 mA, and exposure time of 3000 ms. The disc height was measured using ImageJ software, and the Degenerated Disc Score (DHI%) was calculated according to the previously described method. A Siemens 3T Magnetom Trio Tim scanner (located in Munich, Germany) was used to perform caudal sagittal T2W1 MRI scans of the disc. The disc degeneration was assessed based on the Pfirrmann grading system (Table S4, Supplementary Information).

*Histological evaluation:* The disc samples were fixed in 4% buffered formaldehyde and then decalcified for 1 month with 10% ethylenediaminetetraacetic acid (EDTA; Solarbio, China) before being embedded in paraffin. For histological analysis, 5μm thick sections were prepared and stained with hematoxylin-eosin (H&E) and Safranin O-Fast Green (SOFG), and histological grading was performed (Table S5, Supplementary Information). A score of 5 indicated a normal disc, while a score of 6–11 indicated a moderately degenerated disc, and a score of 12–15 indicated a severely degenerated disc. For tissue immunofluorescence analysis, the tissue sections were fixed and blocked before being incubated overnight at 4 ℃ with primary antibodies GD2 (1:500, BD Pharmingen) and CD24 (1:500, Invitrogen). Then, the secondary antibodies were incubated at room temperature for 1 hour, followed by DAPI staining. Confocal fluorescence microscopy was used for observation, and ImageJ software (NIH, USA) was employed for analysis.

**Supplementary Results:**


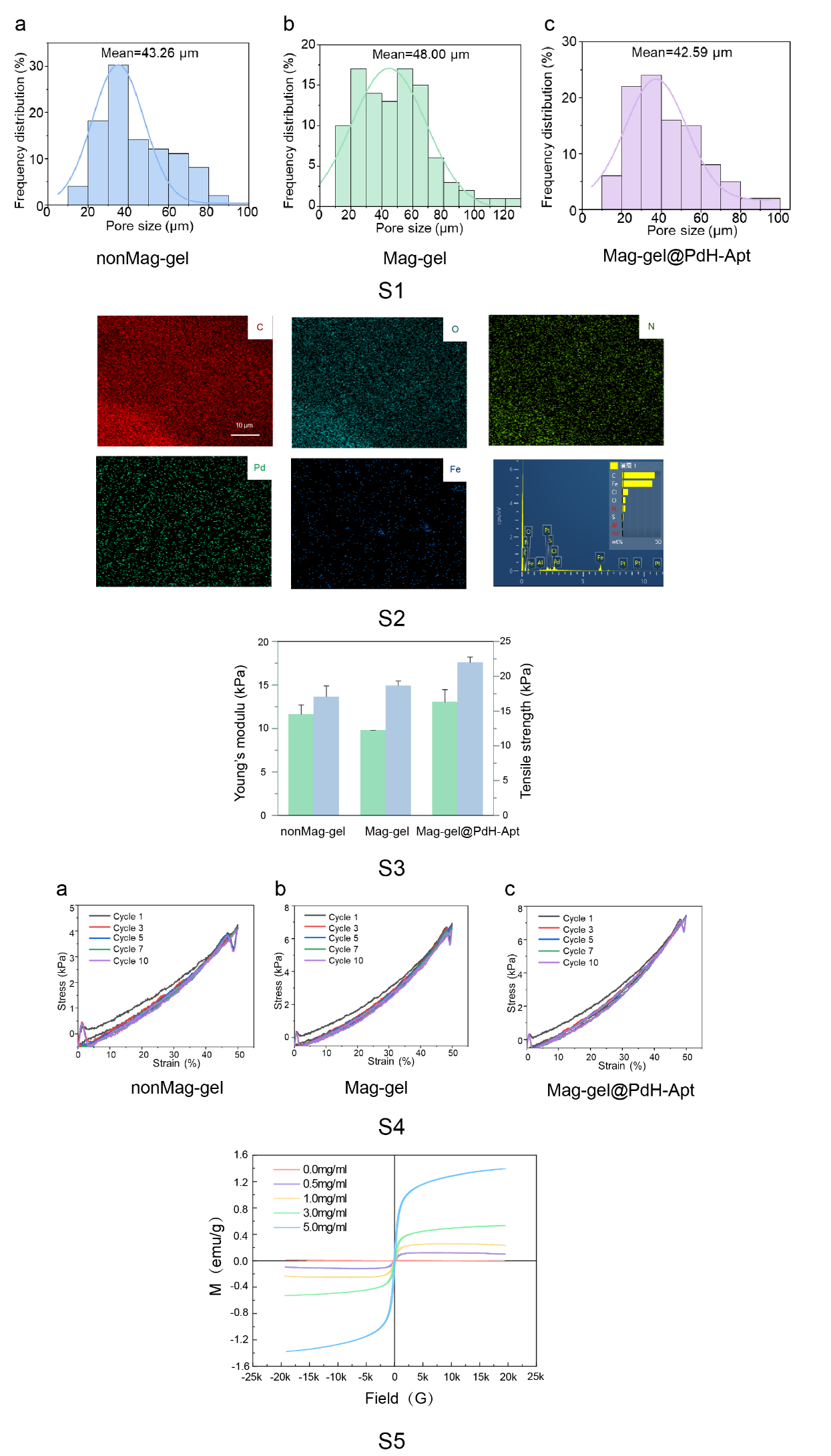


**Figure S1.** Pore size distributions of (a) nonMag-gel, (b) Mag-gel, and (c) Mag-gel@PdH-Apt calculated from TEM images.


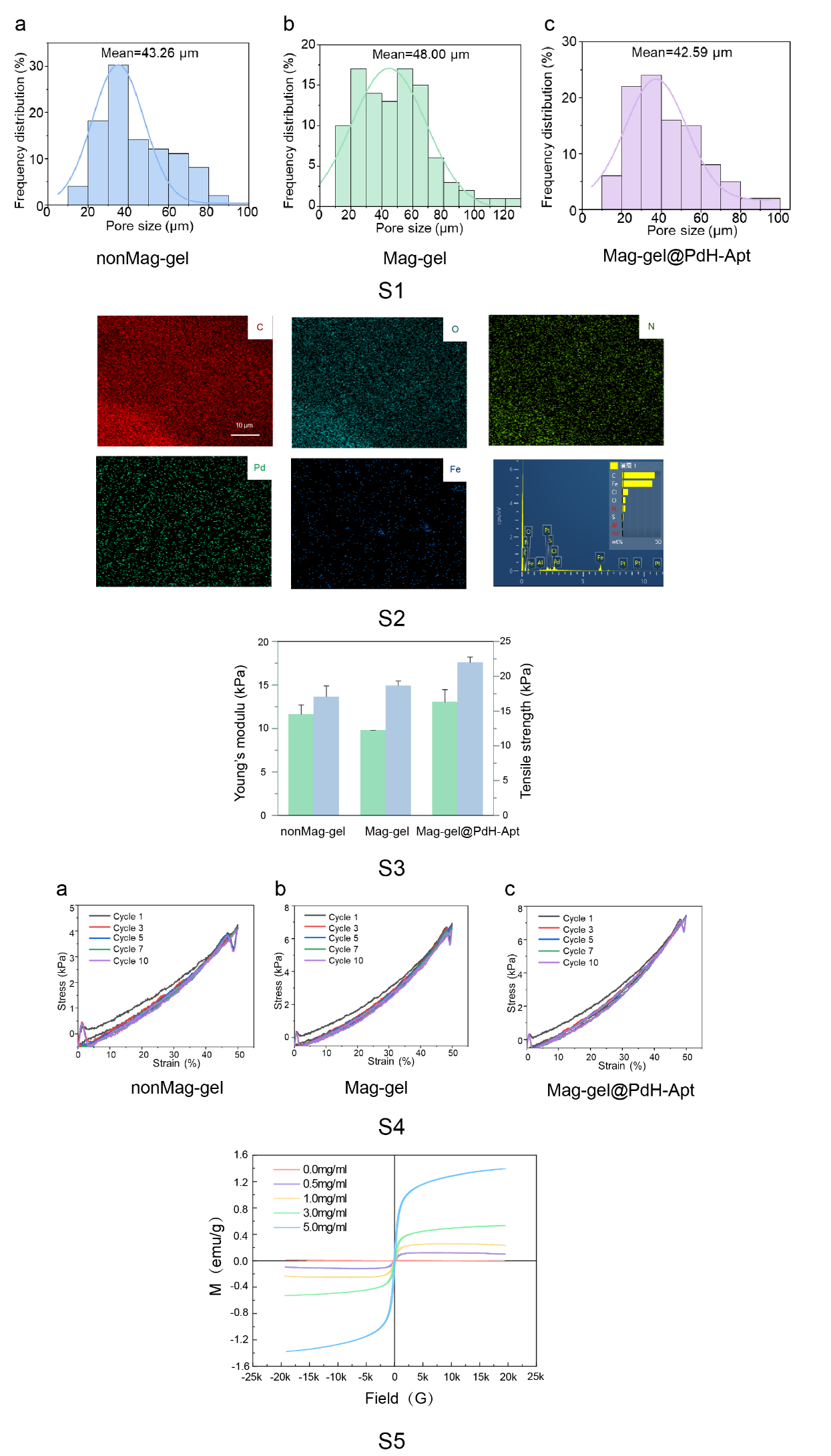


**Figure S2.** Elemental mapping images of Mag-gel@PdH-Apt hydrogel.


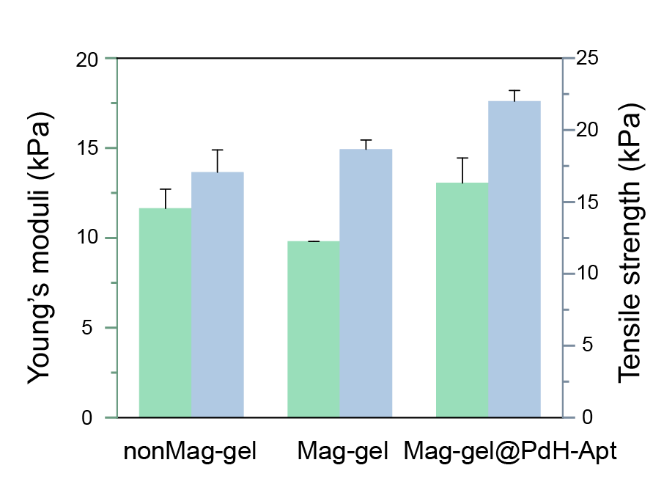


**Figure S3.** Young’s moduli and tensile strengths of nonMag-gel, Mag-gel, and Mag-gel@PdH-Apt.


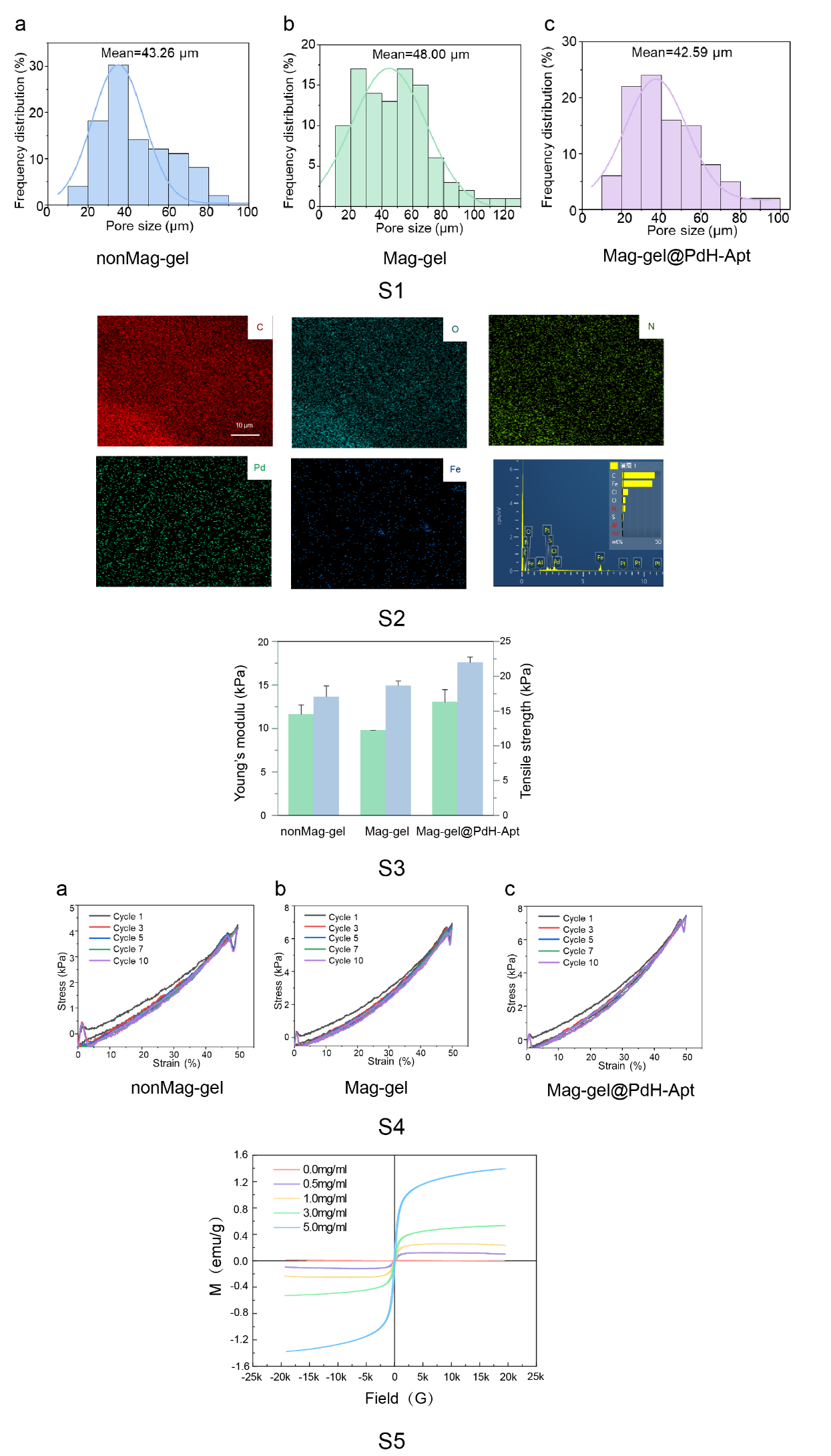


**Figure S4.** Cyclic compression test of (a) nonMag-gel, (b) Mag-gel, and (c) Mag-gel@PdH-Apt.


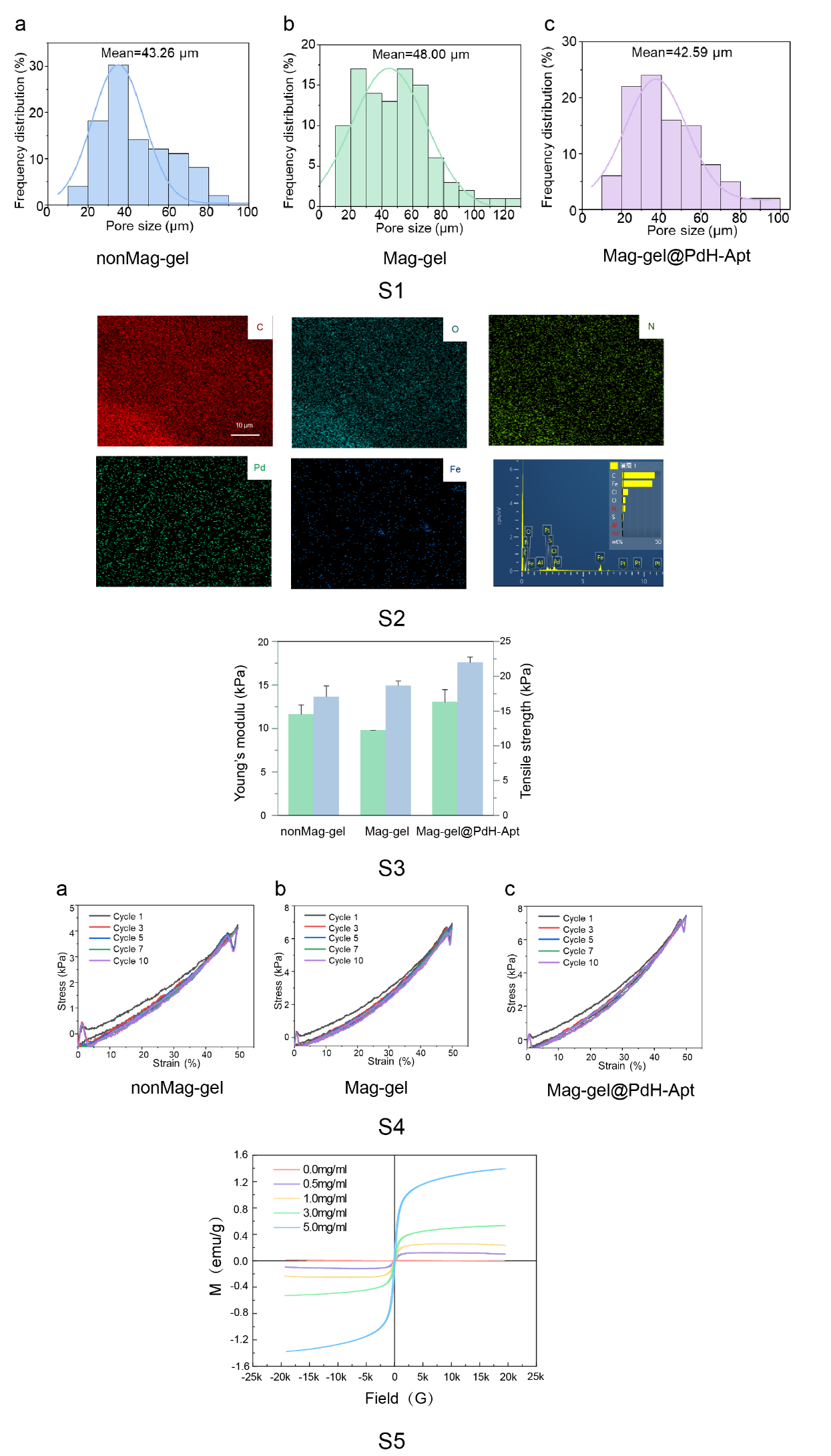


**Figure S5.** Hysteresis loops of Mag-gel with different concentrations of PEG/PEI-SPIONs.


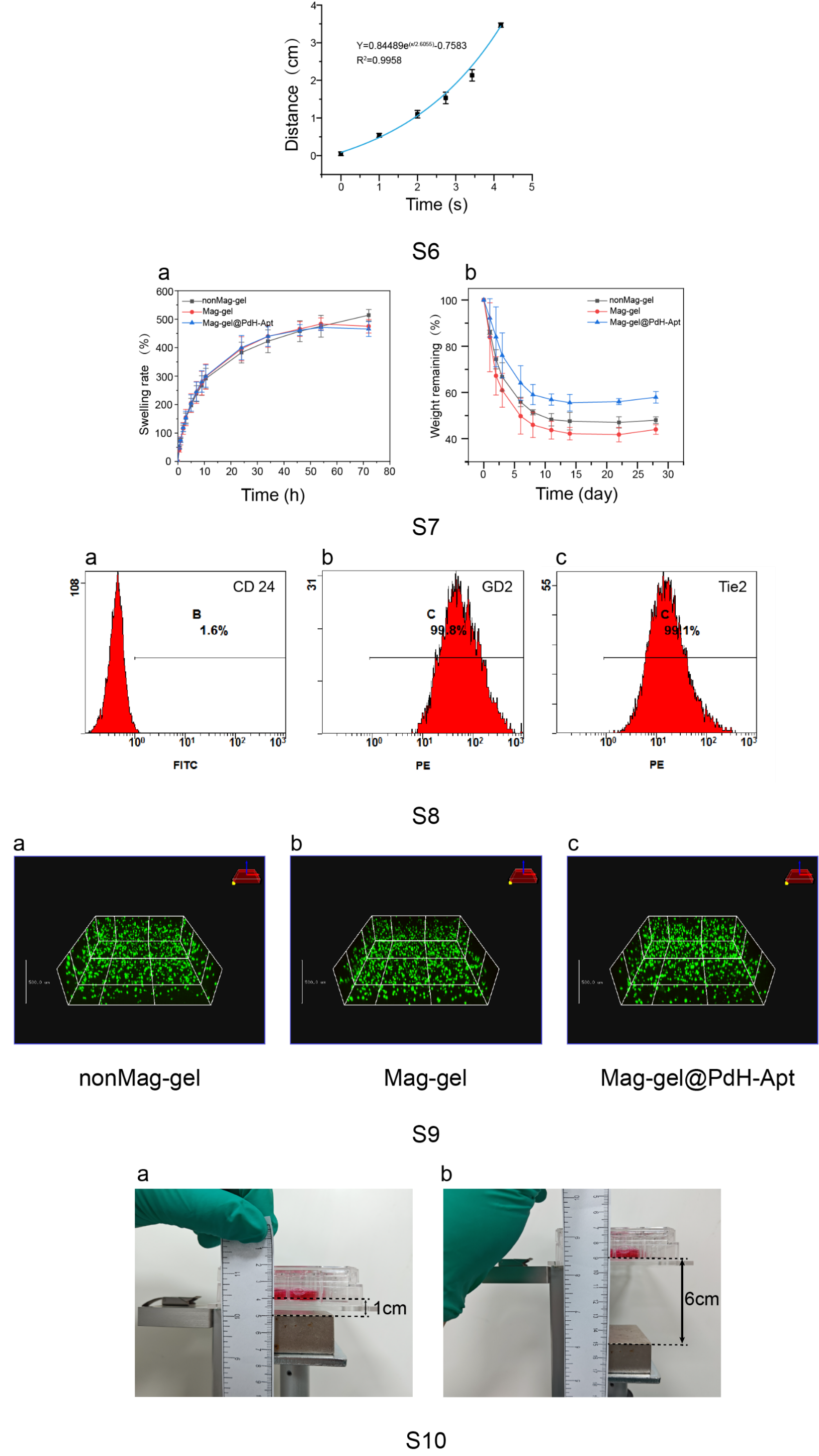


**Figure S6.** Distance *vs* time image of Mag-gel under an attraction of a 1.4 T permanent magnet.


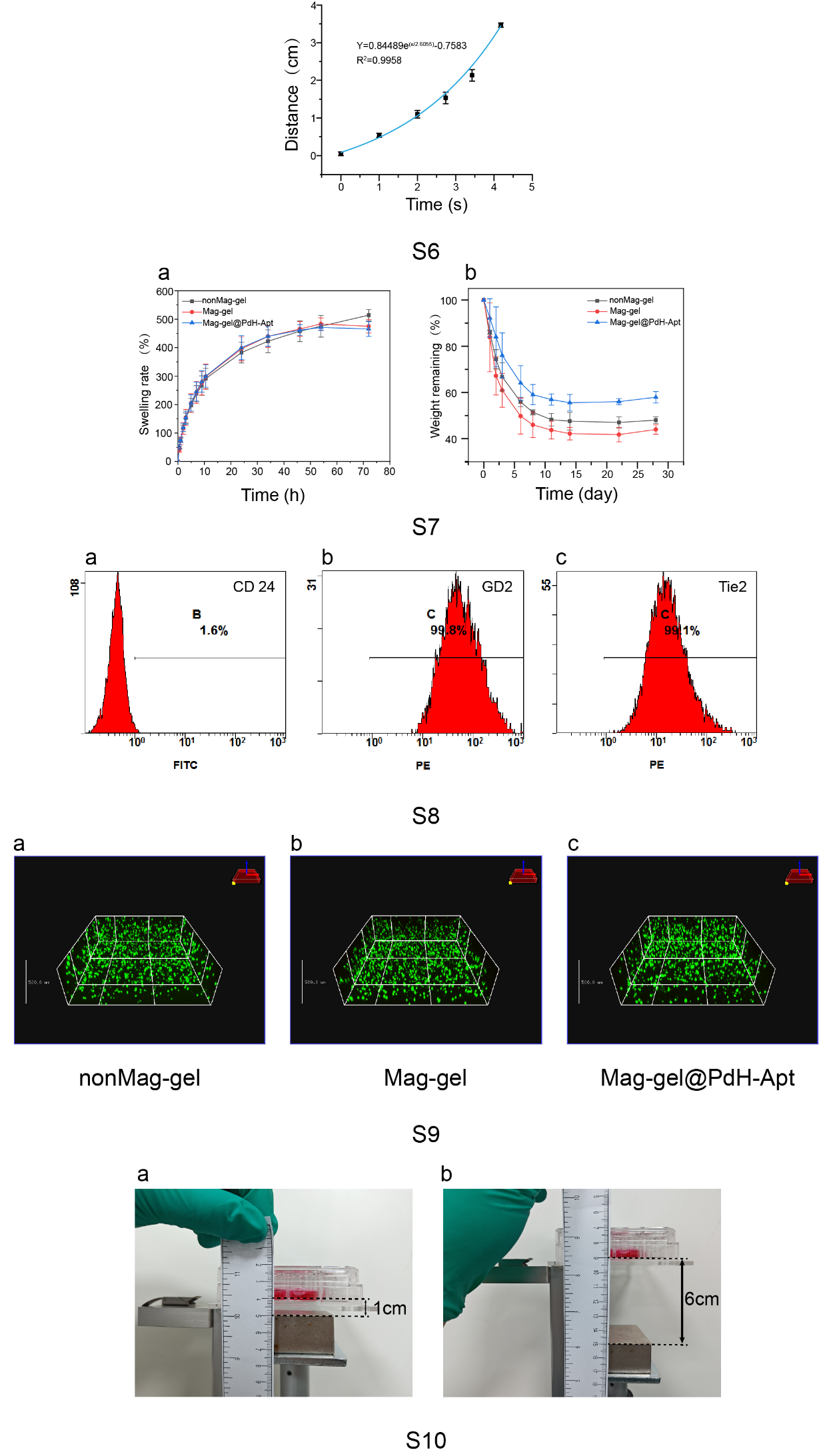


**Figure S7.** Magnetic stimulation device. The closest distance of the permanent magnet to the cells cultured in hydrogel is (a) 1.0 cm, and the farthest distance is (b) 6.0 cm. The total duration of magnetic stimulation is 2 h/day for 7 days.


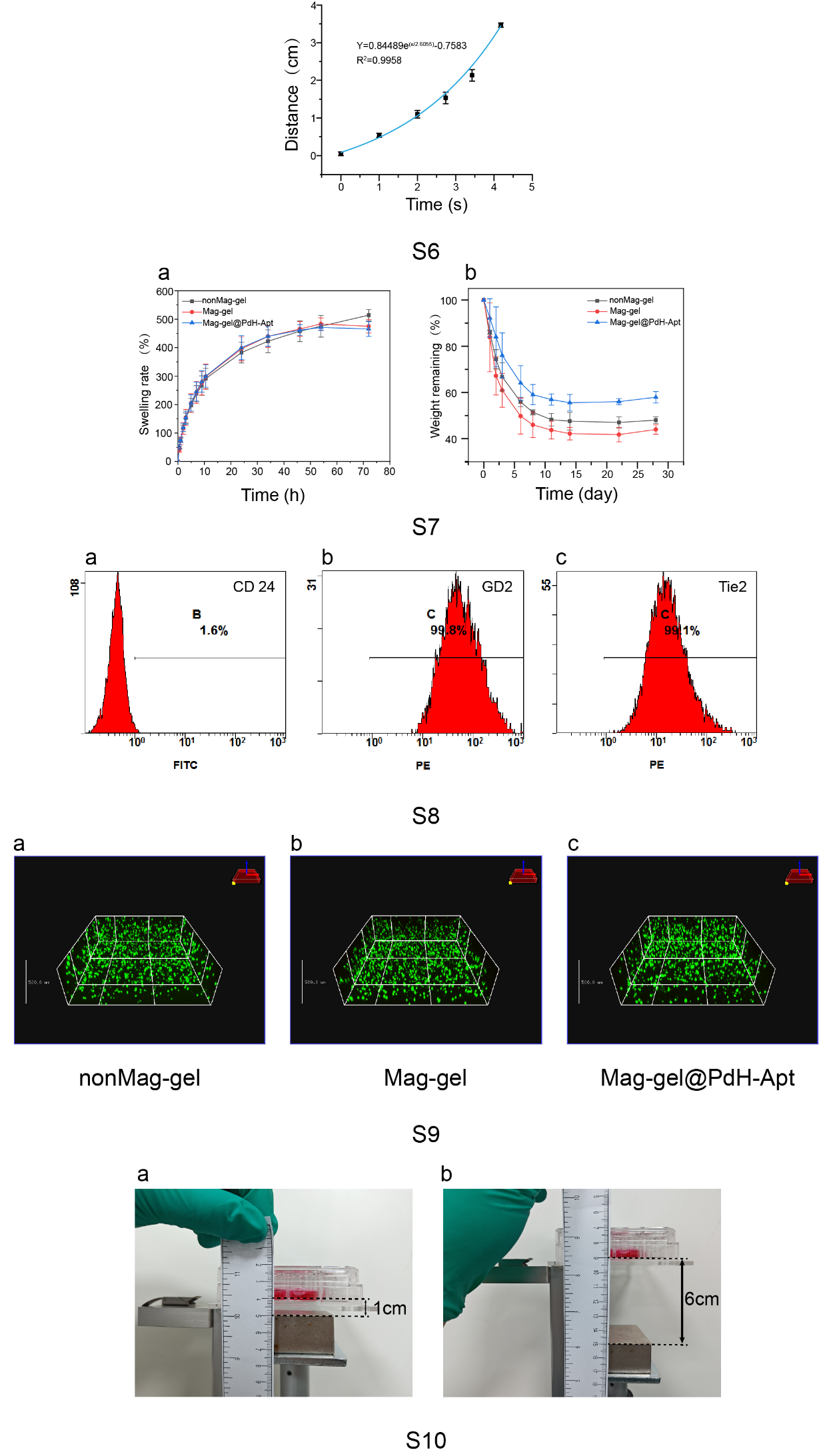


**Figure S8.** (a) Swelling ratios and (b) biodegradation behaviors of nonMag-gel, Mag-gel, and Mag-gel@PdH-Apt hydrogels.


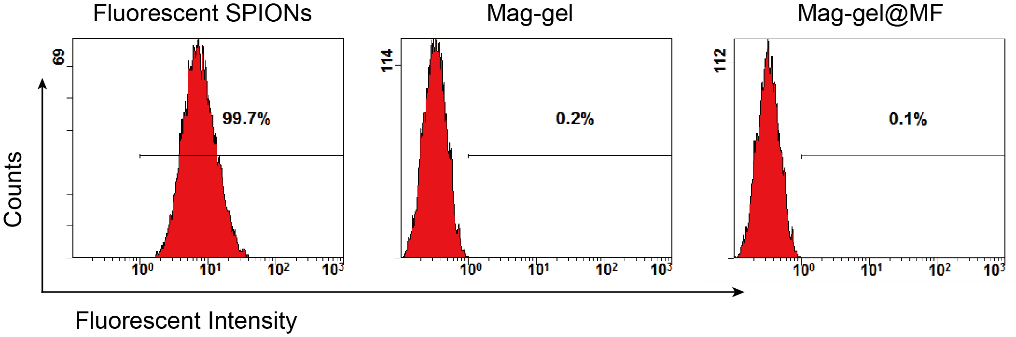


**Figure S9.** Flow cytometric analysis showing negligible leakage of SPIONs from the hydrogel with/without mechanical stimulation.


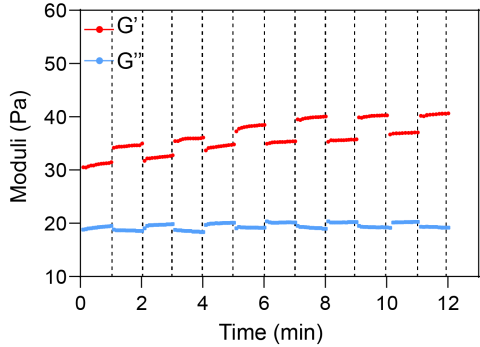


**Figure S10.** Magnetorheological characterization of Mag-gel@PdH-Apt after one-month degradation. Dates are presented as mean ± standard deviation (SD).


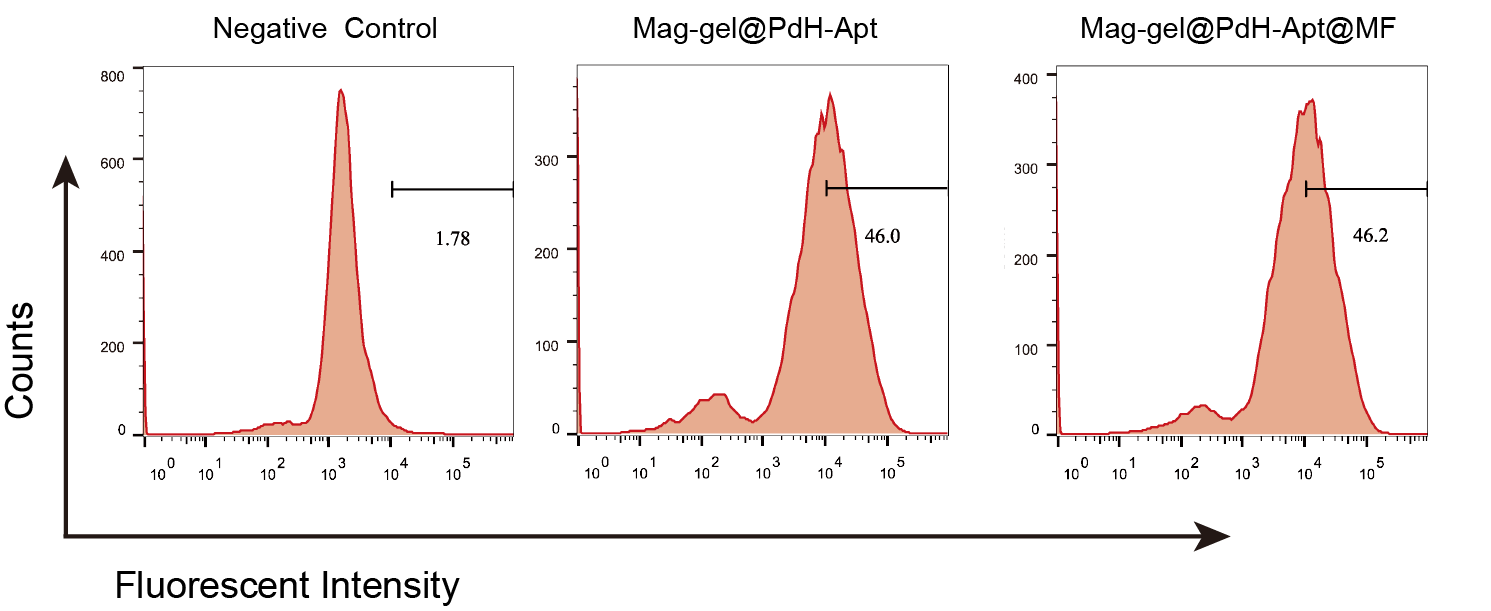


**Figure S11.** Flow cytometric analysis for quantitative assessment of PdH-Apt release ratio after one month of degradation.


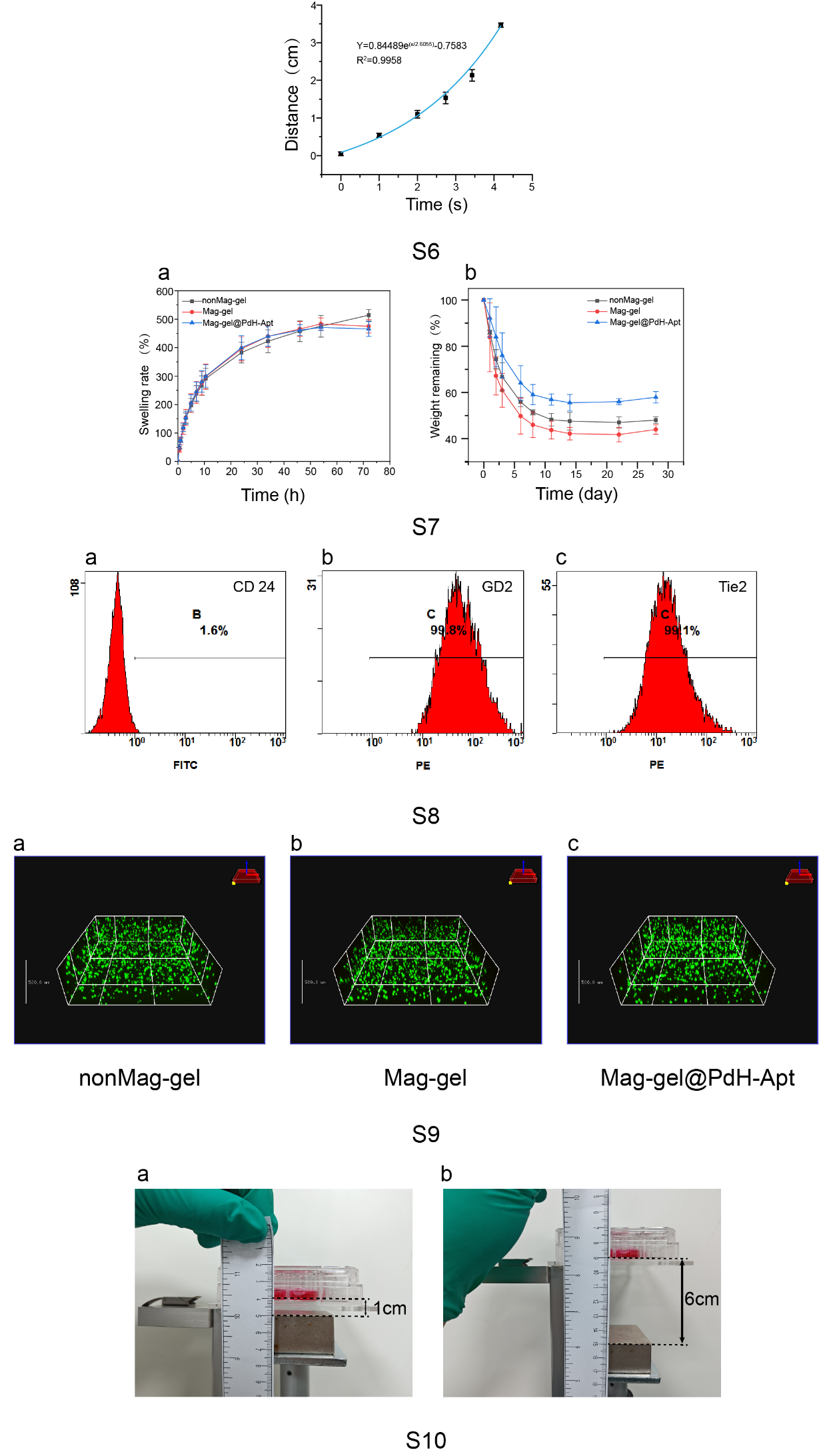


**Figure S12.** Flow cytometry analysis to detect the expression levels of (a) CD24, (b) GD2, and (c) Tie2 proteins on cell surfaces.


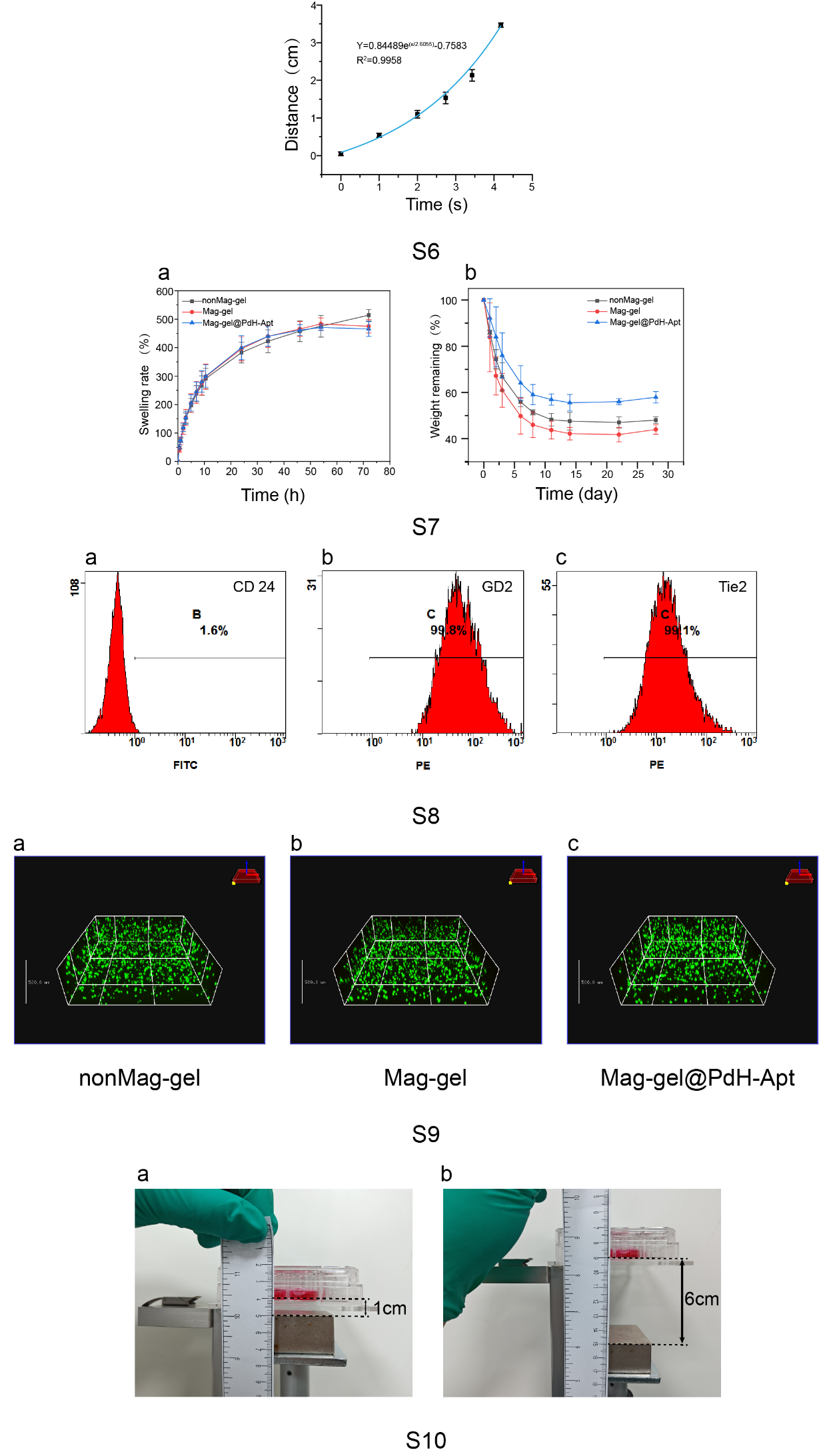


**Figure S13:** Live/Dead staining images of 3D cultured NPSCs in fabricated (a) nonMag-gel, (b) Mag-gel, and (c) Mag-gel@PdH-Apt hydrogels after 72 h of culture *in vitro* (green represented live cells, red represented dead cells).


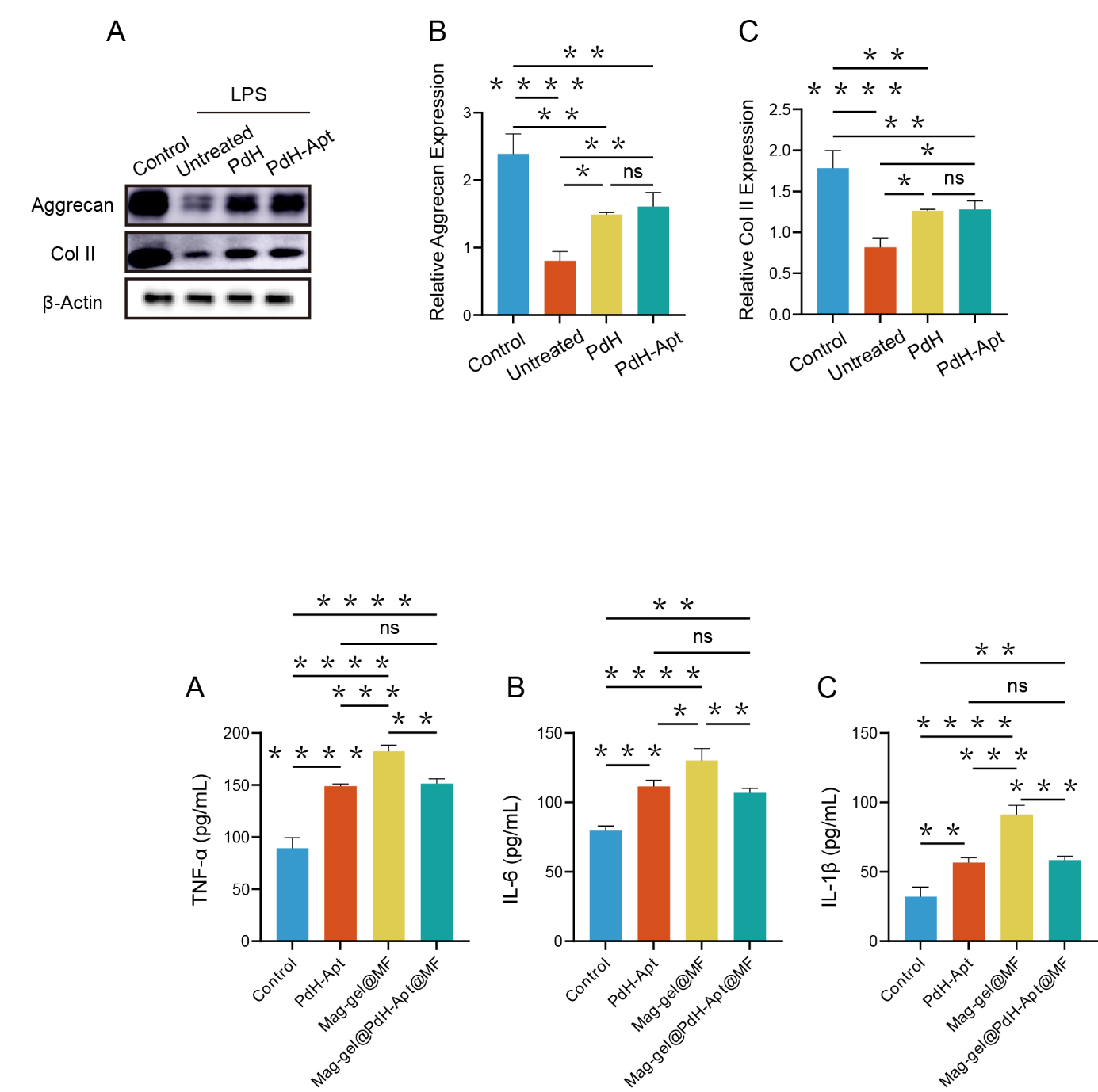
**Figure S14:** (A) Detection of Col II, ACAN and β-actin protein levels in Control, LPS+Untreated, LPS+PdH, and LPS+PdH-Apt groups by WB. Expression levels of (B) Col II, (C) Aggrecan protein in NPSCs across various treatment (n = 3; *P < 0.05, **P < 0.01, and ****P < 0.0001). All statistical data are presented as mean ± SD.


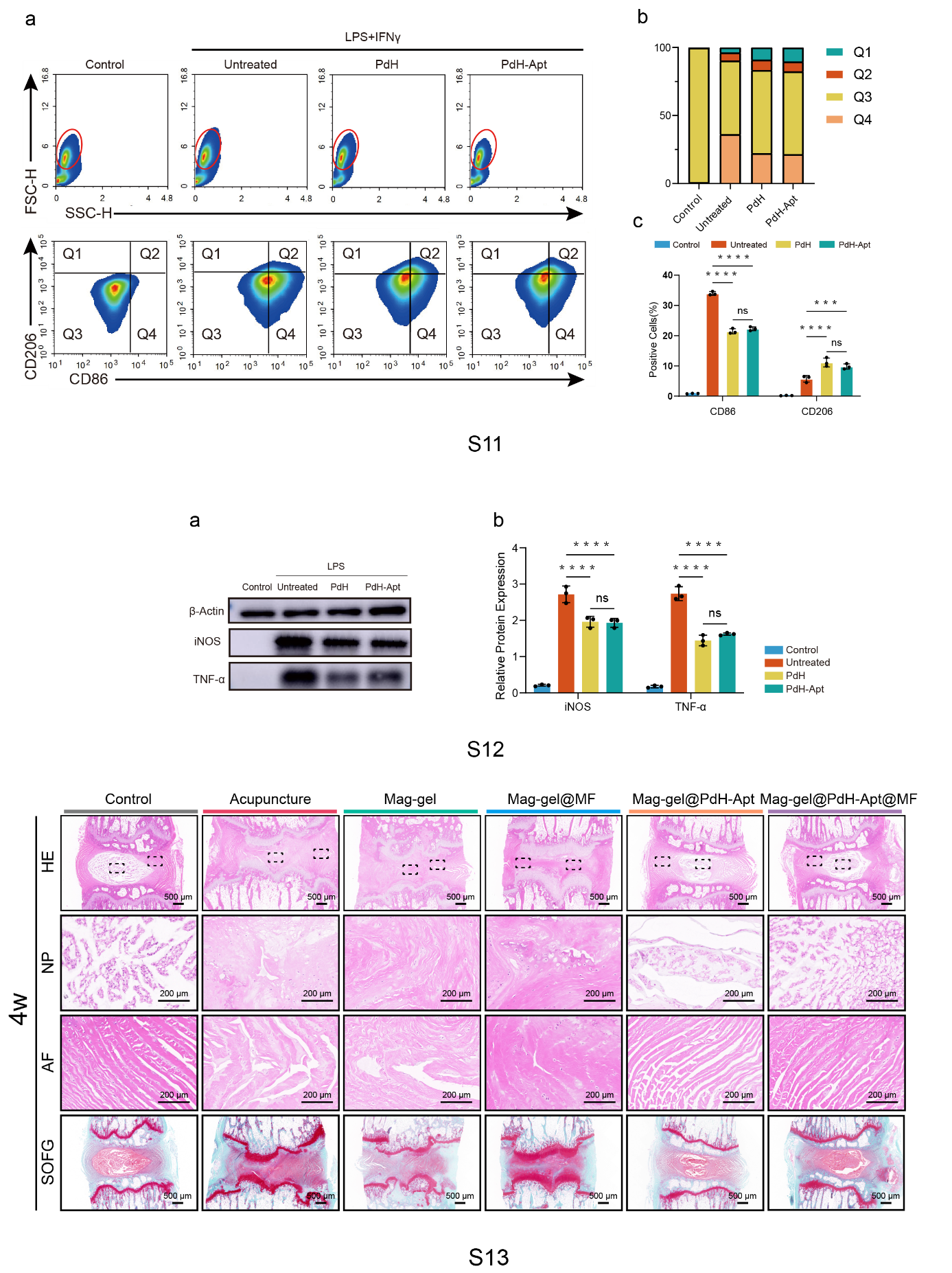


**Figure S15.** Flow cytometry assay and quantification of macrophage polarization. (a) Quadrant plot and (b) statistical analysis of flow cytometry. (c) Proportion of CD86-positive or CD206-positive cells.(*n* = 3, ****P <* 0.001, *****P <* 0.0001). M1 and M2 subtypes were distinguished by the presence of CD86 (mostly in Q3) and CD206 (primarily in Q1). Date are presented as mean ± SD.


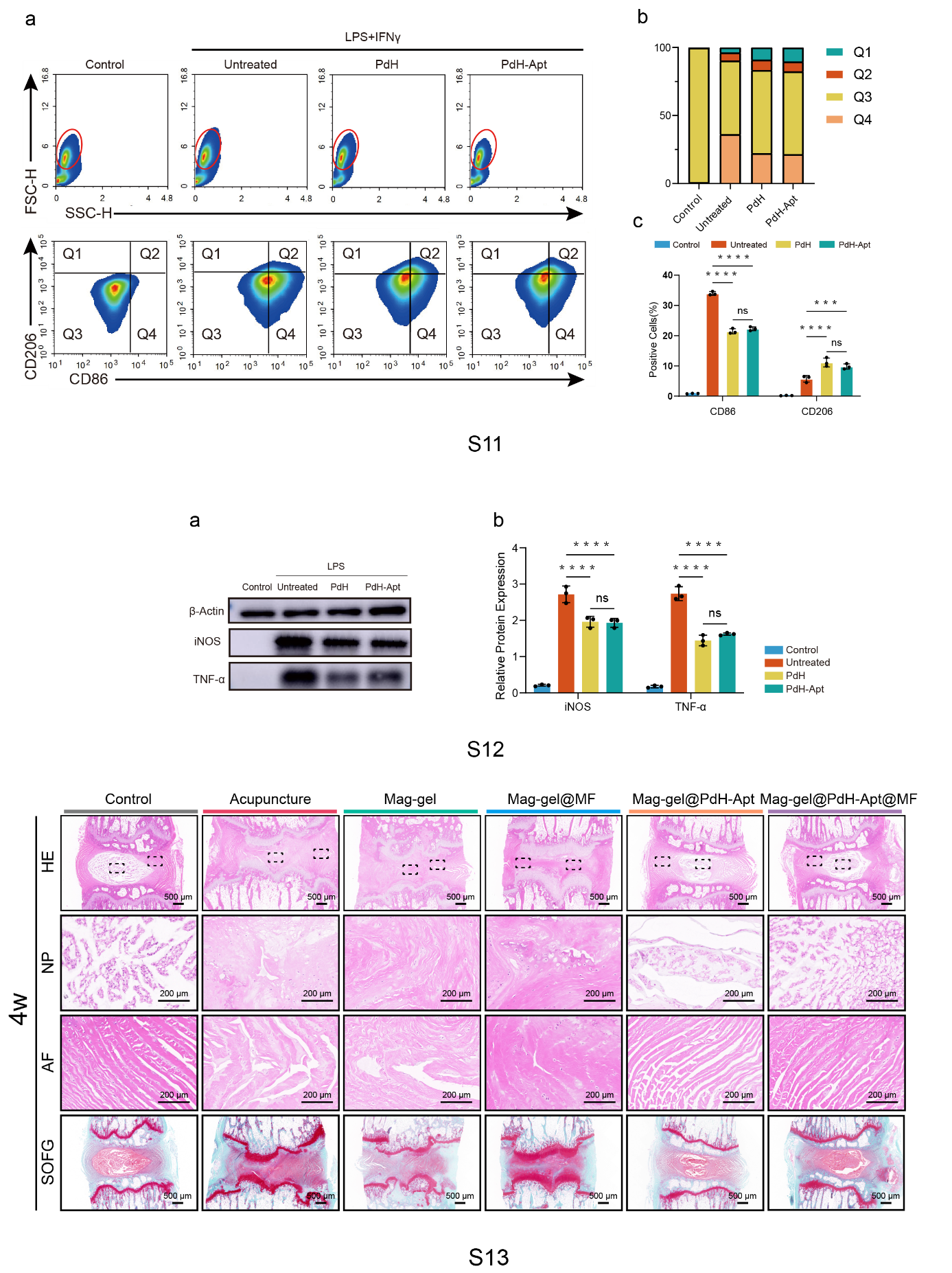


**Figure S16.** (a)Western blotting and (b) quantitative analyses of iNOS and TNF-α expressions in macrophages (*n* = 3, *****P <* 0.0001). Date are presented as mean ± SD.


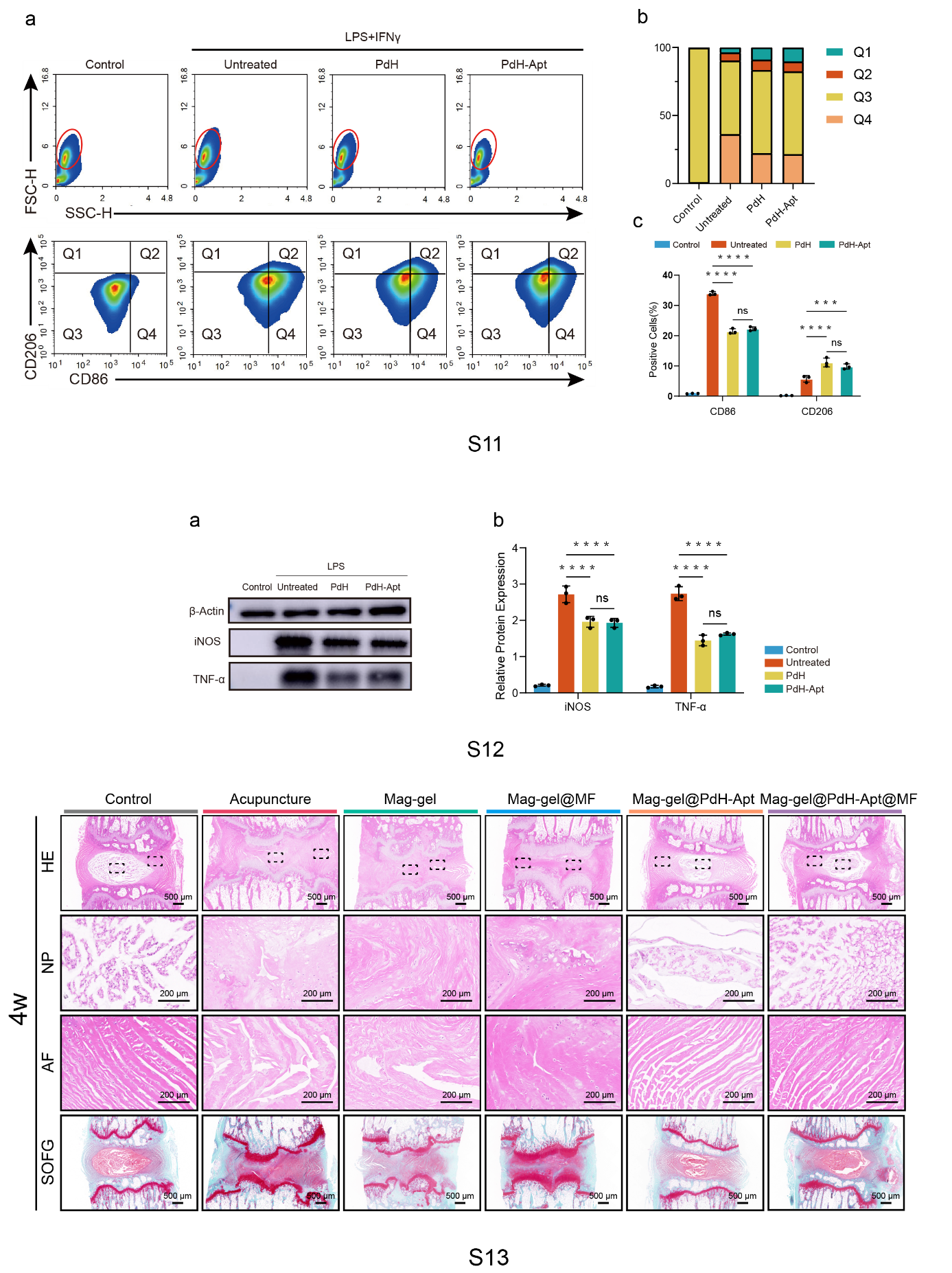


**Figure S17.** H&E and SOFG staining images of IVDs at 4 weeks postoperatively.

**Supplementary Tables:**

**Table S1.** Average time for permanent magnet to complete a cycle of turning.

| Lap | 1 | 2 | 3 | 4 | 5 | 6 | 7 | 8 | 9 | 10 | Average time (frequency) |
| --- | --- | --- | --- | --- | --- | --- | --- | --- | --- | --- | --- |
| Time/s | 9.85 | 10.00 | 9.96 | 10.23 | 10.02 | 9.78 | 10.05 | 10.21 | 9.68 | 10.01 | 9.97 = 0.10 Hz |

**Table S2.** Primer sequences of aptamer DB67.

| Name | Sequence (*5’-3’*) |
| --- | --- |
| DB67 | CCGCCCAAATCCCTAAGAGCCCAAAACAACACAACCACCACACCAC CCAGACACACTACACACGCA |

**Table S3.** Primer sequences used for RT-qPCR analyses in this study.

| Gene | Sequence (forward *5’-3’*) | Sequence (reverse *5’-3’*) |
| --- | --- | --- |
| ACAN | CCTTGGACACTTTCACATGCTTAT | TCCACTGGTAGTCTTGAGCATTT |
| Col2a1 | GAAGAGCGGAGACTACTGGATTG | TCTCCAAACCAGATGTGCTTCTT |
| MMP13 | ACGTGTGGAGTTATGATGATGCT | GCATGACTCTCACAATGCGATTA |

**Table S4.** The data table of inflammation related gene expression.

| Gene name | LPS_1 | LPS_2 | LPS_3 | PdH_Apt_1 | PdH_Apt_2 | PdH_Apt_3 |
| --- | --- | --- | --- | --- | --- | --- |
| Bcl6 | 11.03637 | 10.99835 | 11.04083 | 11.16013 | 11.21954 | 11.08725 |
| Btn1a1 | 5.152019 | 5.662262 | 5.494453 | 5.953216 | 6.232845 | 5.822888 |
| Cd24 | 9.250112 | 9.327214 | 9.244921 | 10.28797 | 10.36141 | 10.33009 |
| Cd44 | 15.31654 | 15.34759 | 15.3682 | 15.43645 | 15.46848 | 15.46848 |
| Ceacam1 | 7.832625 | 7.728213 | 7.95974 | 8.182187 | 8.220797 | 8.190354 |
| Cebpb | 11.38607 | 12.12392 | 11.96839 | 12.96072 | 13.40075 | 13.45687 |
| Fgr | 9.605951 | 9.659024 | 9.515421 | 9.775127 | 9.767034 | 9.832725 |
| Gnrh1 | 7.990211 | 7.998493 | 8.212502 | 8.228432 | 8.385216 | 8.430523 |
| Id2 | 8.238883 | 8.083347 | 8.190354 | 9.280568 | 9.197307 | 9.115227 |
| Loxl3 | 11.39127 | 11.35099 | 11.31999 | 11.71846 | 11.714 | 11.72713 |
| Lrrc32 | 10.23778 | 10.27916 | 10.31791 | 10.65538 | 10.4768 | 10.69404 |
| Pglyrp1 | 7.912692 | 8.038449 | 7.95195 | 8.345536 | 8.185769 | 8.53676 |
| Ripor2 | 7.502474 | 7.507772 | 7.572892 | 7.973251 | 7.785728 | 7.868516 |
| Runx3 | 5.528751 | 5.231196 | 5.603898 | 6.064476 | 6.310649 | 6.58934 |
| Sdc4 | 15.00979 | 14.97894 | 14.93501 | 15.12275 | 15.16095 | 15.18164 |
| Smad7 | 11.73173 | 11.61162 | 11.55567 | 12.15595 | 12.17562 | 12.05981 |
| Spn | 2.321928 | 2.931998 | 4.024664 | 4.559319 | 5.038869 | 4.353182 |
| Tgfb1 | 12.40334 | 12.35835 | 12.39662 | 12.45392 | 12.5272 | 12.60754 |
| Zc3h12d | 5.74001 | 5.603898 | 5.528751 | 6.415069 | 6.349332 | 5.822888 |
| Akap6 | 7.419651 | 7.4517 | 7.292881 | 7.838959 | 7.824303 | 7.983549 |
| Asph | 12.43699 | 12.41091 | 12.45793 | 12.57859 | 12.59989 | 12.52586 |
| Atp1b1 | 11.08491 | 11.0281 | 11.11377 | 11.77459 | 11.75629 | 11.73658 |
| Calm3 | 12.00266 | 11.94655 | 11.89347 | 12.05946 | 12.0923 | 12.07115 |
| Ccl3 | 8.636086 | 8.826385 | 8.562897 | 9.353027 | 9.451024 | 9.299342 |
| F2r | 11.79657 | 11.81888 | 11.80341 | 12.0614 | 11.94064 | 12.00755 |
| Fgf14 | 1 | 0 | 1.584963 | 2.696159 | 1.584963 | 2.365767 |
| Hap1 | 8.234961 | 8.17078 | 8.340026 | 8.833083 | 8.748647 | 8.748647 |
| Pla2g1b | 1 | 0 | 0 | 2.696159 | 2.696159 | 1.584963 |
| Plcg1 | 12.54925 | 12.50758 | 12.54402 | 12.685 | 12.59253 | 12.685 |
| Ramp3 | 11.75452 | 11.64938 | 11.62501 | 12.18296 | 12.21416 | 12.2108 |
| Serpine1 | 17.77938 | 17.77938 | 17.77938 | 18.17947 | 18.17947 | 18.26492 |
| Stac | 6.993047 | 6.708682 | 6.8628 | 7.778134 | 7.80937 | 8.011508 |
| Stac2 | 8.978766 | 8.896517 | 8.841709 | 9.991799 | 10.10941 | 10.10333 |
| Stc1 | 11.93607 | 11.91743 | 11.97612 | 12.33042 | 12.24906 | 12.33213 |
| Thy1 | 13.55641 | 13.52779 | 13.48676 | 13.70618 | 13.80818 | 13.74611 |
| Trpc1 | 8.362396 | 8.553931 | 8.397959 | 8.836251 | 8.941668 | 8.917376 |
| Tspo | 12.7675 | 12.67282 | 12.59212 | 12.89901 | 12.86518 | 12.91417 |
| Acp5 | 10.30651 | 10.28116 | 10.21402 | 11.09322 | 10.94459 | 11.03874 |
| Appl2 | 10.51396 | 10.5573 | 10.55647 | 10.75009 | 10.83076 | 10.79661 |
| Cd200 | 12.44678 | 12.52586 | 12.46911 | 12.8372 | 12.80777 | 12.86725 |
| Cd200r1 | 6.116767 | 6.197315 | 5.868936 | 6.931405 | 7.100591 | 6.758174 |
| Gata3 | 5.437672 | 5.010445 | 5.753261 | 6.137487 | 5.74001 | 6.042677 |
| Gpx1 | 12.54556 | 12.54294 | 12.42962 | 12.85437 | 12.80613 | 12.81229 |
| Hgf | 10.39744 | 10.41704 | 10.4998 | 10.63919 | 10.65755 | 10.54545 |
| Ier3 | 14.71061 | 14.64287 | 14.66215 | 14.83562 | 14.84394 | 14.82118 |
| Metrnl | 10.34389 | 10.37355 | 10.23451 | 10.46999 | 10.48979 | 10.51806 |
| Nr1d1 | 10.78031 | 10.76646 | 10.89049 | 11.1406 | 11.25576 | 11.29529 |
| Nt5e | 8.676442 | 8.622604 | 8.539264 | 9.576959 | 9.461958 | 9.466381 |
| Pbk | 7.56351 | 7.616081 | 7.562322 | 8.081551 | 8.010638 | 8.23805 |
| Pycard | 5.96824 | 5.961008 | 5.921222 | 6.429284 | 6.248513 | 6.429284 |
| Rora | 8.088672 | 8.195299 | 8.10923 | 8.40477 | 8.385216 | 8.486744 |
| Sirpa | 10.83618 | 10.74207 | 10.84675 | 10.96832 | 10.95455 | 10.98362 |
| Tnfrsf1b | 10.73181 | 10.73223 | 10.83433 | 11.24902 | 11.00834 | 10.96748 |
| Uaca | 11.77216 | 11.75766 | 11.7834 | 11.93113 | 11.96584 | 12.00541 |
| Wfdc1 | 8.385879 | 8.262762 | 8.223599 | 8.908083 | 8.62446 | 8.586236 |
| Zfp36 | 10.50121 | 10.41006 | 10.48032 | 10.78568 | 10.78866 | 10.83606 |

**Table S5.** Pfirrmann disc degeneration score.

|  | Ⅰ | Ⅱ | Ⅲ | Ⅳ | Ⅴ |
| --- | --- | --- | --- | --- | --- |
| Structure | Homogeneous, bright white | Inhomogeneous with or without horizontal bands | Inhomogeneous, gray | Inhomogeneous, gray to black | Inhomogeneous, black |
| Distinction of Nucleus and Anulus | Clear | Clear | Unclear | Lost | Lost |
| Signal Intensity | Hyperintense, isointense to cerebrospinal fluid | Hyperintense, isointense to cerebrospinal fluid | Intermediate | Intermediate to hypointense | Hypointense |
| Height of Intervertebral disc | Normal | Normal | Normal to slightly decreased | Normal to moderately decreased | Collapsed disc space |

**Table S6.** Histological grade scale of intervertebral disc.

| Ⅰ. Nucleus pulposus morphology  Grade:  0: round shape, nucleus pulposus > 75% of disc area  1: round shape, nucleus pulposus = 50%−75% of disc area  2: nucleus pulposus = 25%−50% of disc area  3: nucleus pulposus < 25% of disc area | Ⅱ. Nucleus pulposus cellularity  Grade:  0: stellar-shaped cells  1: most stellar-shaped cells with some round cells  2: most round cells with some stellar shaped cells  3: round-shaped cells |
| --- | --- |
| Ⅲ. Annulus fibrosus morphology  Grade:  0: well-organized lamellae with no ruptures  1: ruptured fibers < 25% of annulus fibrosus  2: ruptured fibers = 25%−50% of annulus fibrosus  3: ruptured fibers > 50% of annulus fibrosus | IV. Annulus fibrosus cellularity  Grade:  0: fibroblasts > 90% of cells  1: fibroblasts = 75%−90% of cells  2: fibroblasts = 25%−75% of cells  3: fibroblasts < 25% of cells |
| V. Border between nucleus pulposus and annulus fibrosus  Grade:  0: normal, without any interruption  1: minimal interruption  2: moderate interruption  3: severe interruption |  |
